# Marangoni effect inspired robotic self-propulsion over a water surface using a flow-imbibition-powered microfluidic pump

**DOI:** 10.1038/s41598-021-96553-8

**Published:** 2021-09-01

**Authors:** Bokeon Kwak, Soyoung Choi, Jiyeon Maeng, Joonbum Bae

**Affiliations:** Bio-Robotics and Control (BiRC), Department of Mechanical Engineering, Ulsan National Institute of Science and Engineering (UNIST), Ulsan, 44919 Republic of Korea

**Keywords:** Mechanical engineering, Surface chemistry, Materials for devices

## Abstract

Certain aquatic insects rapidly traverse water by secreting surfactants that exploit the Marangoni effect, inspiring the development of many self-propulsion systems. In this research, to demonstrate a new way of delivering liquid fuel to a water surface for Marangoni propulsion, a microfluidic pump driven by the flow-imbibition by a porous medium was integrated to create a novel self-propelling robot. After triggered by a small magnet, the liquid fuel stored in a microchannel is autonomously transported to an outlet in a mechanically tunable manner. We also comprehensively analyzed the effects of various design parameters on the robot’s locomotory behavior. It was shown that the traveled distance, energy density of fuel, operation time, and motion directionality were tunable by adjusting porous media, nozzle diameter, keel-extrusion, and the distance between the nozzle and water surface. The utilization of a microfluidic device in bioinspired robot is expected to bring out new possibilities in future development of self-propulsion system.

## Introduction

**Figure 1:**
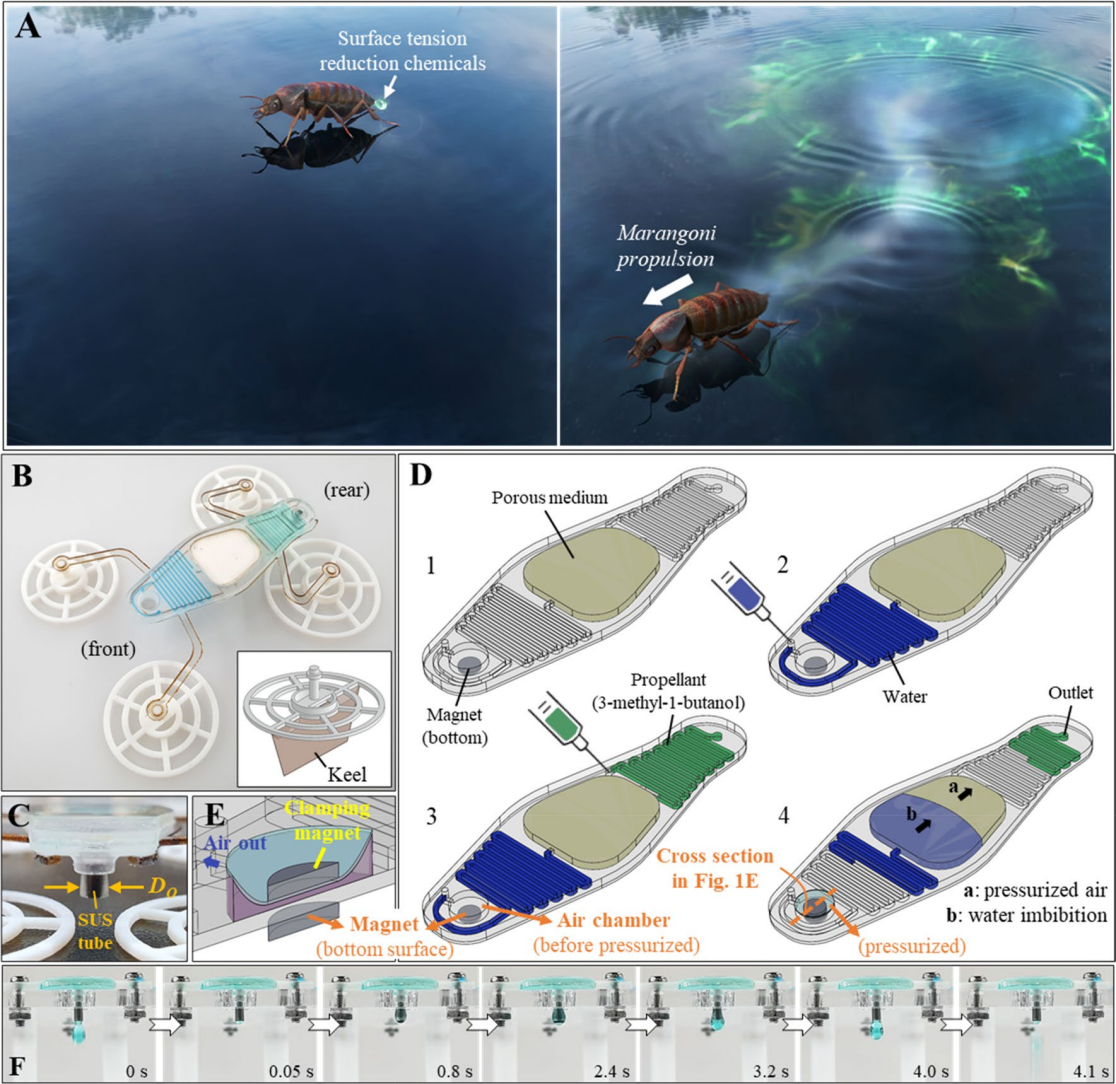
Structure and working principle of the robot exploiting Marangoni propulsion. (**A**) Secretion of chemicals that reduce surface tension. (**B**) The proposed self-propelling robot (bottom-right corner: a keel-extruded footpad). (**C**) The rear side of the robot. (**D**) The preparative steps prior to pump activation. (**E**) A cross-section of the air-filled chamber of D-(step 4). (**F**) Alcohol droplet formation during pump operation (*D*_*O*_ = 1.5 mm).

The characteristics of bio-locomotion on the air/water interface is different from those of other environments; surface tension plays an important role. Arthropods such as the rove beetles *Microvelia* and *Stenus* are both supported by water surface tension, and rapidly (up to 70 cm/s) skim over the water surface as shown in Fig. 1A by secreting chemicals that momentarily reduce the surface tension to the rear^1–3^. Such locomotion is commonly known as Marangoni propulsion, caused by interfacial flow toward increasing surface tension, and has been widely used in the development of self-propelling systems (Table 1)^4–6^. Alcohol commonly serves as the propellant; however, other organic compounds are sometimes used. Various methods of propellant delivery to the water surface have been described, of which spontaneous transfer via a propellant-infused matrix is the most common^7–14^, although thrust is sometimes generated by dispensing alcohol droplets^15–18^. Spontaneous release of alcohol from a reservoir is also employed^19–23^. Other interesting mechanisms include vapor diffusion^24^, alcohol condensation^25^, depolymerization reactions^26^, and photothermally induced fuel release^27^.

**Table 1:** State-of-the-art in self-propulsion systems driven by Marangoni effect.

Mechanism	Trigger	Refs.
Propellant transfer from soaked gel, droplet, pellet, or other forms of matrix	No	^7–14^
Dispensing alcohol droplets by own weight	No	^15^
	Servo motor	^16,17^
	Water droplet	^18^
Spontaneous release from a reservoir	No	^19–22^
	Alkaline solution	^23^
Vapor diffusion	No	^24^
Condensation	No	^25^
Depolymerization reaction	No	^26^
Photo-thermally induced alcohol release	Near-infrared (NIR) laser	^27^
Self-powered microfluidic pump	Magnetic clamping	This work

Here, no trigger means the self-propulsion started immediately when it was placed on liquid/air interface, or right after filling with propellant.

Many such systems trigger immediate propulsion, and some studies have sought to control the timing of thrust generation (Table 1)^16–18,23,27^. However, the actuators increase weight^16,17^, and speed is slowed at certain pH values^23^. Also, some triggers did not act instantly^18,27^. An additional problem associated with droplet-based dispensing is the need for a vertical vessel filled with propellant that flows out under its own weight^15–18^. A fully loaded vessel concentrates force on the rear of the robot and increases the risk of submergence during propulsion.

Here, we develop a new Marangoni propulsion-based system: a robot that travels on water featuring a capillary pressure-driven microfluidic pump as shown in Fig. 1B. Capillary pressure induced by liquid imbibition by a porous medium has previously been exploited to drive paper-based microfluidic devices^28–30^. However, such pump was redesigned to develop our new self-propulsion system. We use magnetic clamping to trigger the formation of alcohol droplets at required times. Passive actuation is required, and motion is instantaneous, pH-independent, and repeatable. The proposed pump is comprehensively analyzed by adjusting its outlet diameter and embedded porous medium. We also performed a full locomotion analysis and compared the robot to previous systems. Given the recent advances in soft machines featuring autonomous flow regulation^31,32^, microfluidic self-propulsion systems will find many applications.

## Results

### The working principle of the robot

The robot features four footpads allowing it to float by exploiting surface tension (Fig. 1B)^33^. The rear footpads are larger than the front footpads because the alcohol droplets reduce the surface tension at the rear. The maximal supported force by all the four footpads is about threefold that of the robot including fuel mass (refer Supplementary Note: Maximal supporting force). A footpad can (optionally) feature a thin keel (Fig. 1B, bottom-right corner) for directional motion (refer Supplementary Note: Drag asymmetry). Microchannels filled with blue-dyed water and green-dyed alcohol are located at the front and rear of the pump, respectively. A stainless-steel (SUS) tube of outer diameter *D*_*O*_ is located to the rear of the pump (Fig. 1C) and generates alcohol droplets when the pump is operating (Fig. 1D). A porous medium is placed in the center of the pump and the top sealed (step 1). Water is injected into the channel (step 2), followed by alcohol (step 3). We used 3-methyl-1-butanol (3Me1Bu) because the Marangoni effect thereof is prolonged^34^. It is important to ensure that the apices of the injected liquid streams do not contact the porous medium. Note that a magnet is readily attached to the bottom surface of the air chamber before activating the pump. Finally, another magnet (i.e. clamping magnet) is manually placed on the air chamber to trigger water imbibition (step 4); the attractive force between the two magnets instantly pressurizes the air chamber and its cross section is given in Fig. 1E. During water imbibition, air within the porous medium becomes pressurized and pushes the alcohol out via the nozzle (Fig. 1F; Supplementary Videos S1–S4). The alcohol droplets fall near the rear footpads and the robot moves by Marangoni effect. The pump operates until all the water is fully imbibed by the porous medium. By comparing the pumping speed of propellant and the successful rate of magnetic clamping (refer Supplementary Note: Porous media selection and Table S1)^35,36^, two types of porous media (MN 617 filter paper [FP] and cellulose powder [CP]) were selected and used in the rest of the experiments. Please refer Table 2 for the definitions of parameters and acronyms used in this article.

**Table 2:** The definitions of all the parameters and acronyms used on the main text.

Symbols	Definitions	Symbols	Definitions
*D*_*O*_	Nozzle diameter	*σ*_*a*_	Surface tension of alcohol
FP	Filter paper	*σ*_*w*_	Surface tension of water
CP	Cellulose powder	BTB	Bromothymol blue
*h*_*f*_	Footpad height	*R*_*c*_	Ring trace radius
*Q*_*w*_	Average flow rate of water	*S*_*d*_	Traveled distance
*Q*_*a*_	Average flow rate of alcohol	*ε*_*K*, max_	Energy density of fuel
*P*_*c*_	Capillary pressure	*T*_*m*_	Duration of motion
*ϕ*	Porosity	*C*_*θ*_	Trajectory characteristic
*r*_*m*_	Mean pore radius	*m*_robot_	Mass of the robot
*θ*	Contact angle	*m*_fuel_	Mass of the fuel
*k*	Permeability	*V*_max_	Maximum propulsion speed
*V*_*a*_	Droplet volume of alcohol	Δ*T*	Sampling time in *C*_*θ*_
*M*_*a*_	Total volume of injected alcohol	*θ*_*i*_	Heading angle change (*i* = 1, 2, …)
*T*_drop_	Time between breakup of two consecutive droplets	*T*_*aw*_	Time between two consecutive alcohol–water surface contact
*N*_drop_	Total number of droplets generated	*h*_*a*_	Oscillation of alcohol droplet height
*g*	Gravitational acceleration constant	*We*_max_	Maximum Weber number
*ψ*	Harkins-Brown (H-B) correction factor	*Re*_max_	Maximum Reynolds number
*ρ*_*a*_	Density of alcohol	*w*_*f*_	Foot width (or, characteristic length)
*ρ*_*w*_	Density of water	fps	Frames per second
*μ*_*w*_	Kinematic viscosity of water	*n*	The number of trials

### Characteristics of the proposed pump

Dripping dynamics are affected by the liquid per se, the outlet nozzle diameter, and the flow rate. We tested nozzles of diameter *D*_*O*_ = 1.0, 1.5, 2.2, and 3.2 mm, and adjusted the flow rate by varying the porous medium. We measured droplet counts, dripping intervals, and periodicity. The volumes of injected water and alcohol were around 0.13 mL as summarized in Supplementary Table S2.

**Figure 2:**
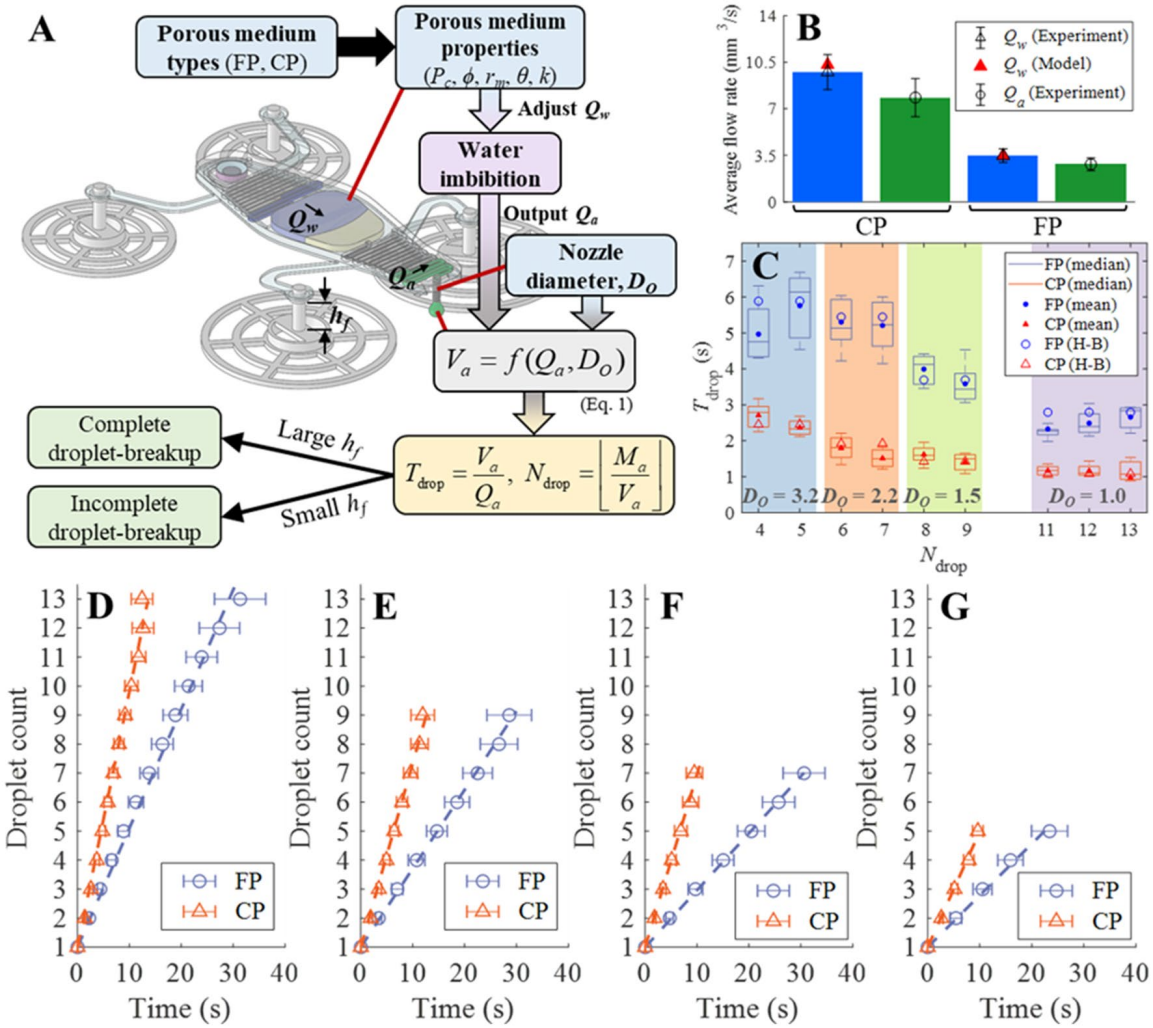
Analysis of alcohol dripping. (**A)** The relations from porous medium types to droplet breakup. Please refer Table 2 for the definitions of parameters and acronyms. (**B**) Average flow rates of water and alcohol (*n* = 5). (**C**) *T*_drop_ vs. *N*_drop_ during the pump operation where *D*_*O*_ denotes the nozzle diameter in millimeter (*n* = 15). Theoretical *T*_drop_ values incorporating the Harkins-Brown (H-B) correction factors are depicted by hollow circles. (**D**–**G**) The elapsed time of alcohol droplet breakup (**D**: *D*_*O*_ = 1.0, **E**: *D*_*O*_ = 1.5, **F**: *D*_*O*_ = 2.2, **G**: *D*_*O*_ = 3.2 mm); dashed lines represent linear regression data (*n* = 15). Note that all the error bars denote standard deviations.

The key parameters were porous medium type and nozzle diameter. As it will be shown in locomotion analysis, two additional parameters (i.e. footpad height and keel-extrusion) will be considered. Here, Fig. 2A shows how the porous medium types affect droplet breakup with other properties such as nozzle diameter and footpad height. Each porous medium exhibit different properties as summarized in Table 3, and their identification methods are described in Supplementary Notes: Capillary pressure measurement^37^, Porosity measurement, and Contact angle and mean pore radius. Average flow rate of water (*Q*_*w*_) and the resultant alcohol flow rate (*Q*_*a*_) depend on porous medium as suggested in Fig. 2B; the flow rates were almost the same in different nozzle diameters (Supplementary Notes: Average flow rates)^38^. In this paper, the number of trials is denoted as *n*. Notably, *Q*_*a*_ was about 80% of *Q*_*w*_ for both FP and CP, likely attributable to (a very small extent of) air leakage during pumping, and pressure transmission loss from the frontal interface of the imbibed water to the alcohol. Also, *Q*_*w*_ is well explained by its model (see Supplementary Notes: Flow rate models for details)^39–46^. Both *Q*_*a*_ and *D*_*O*_ determine an alcohol droplet volume (*V*_*a*_) which found as:

1 $$V_{a} = \psi \left[ {\frac{{\pi D_{O} \sigma_{a} }}{{\rho_{a} g}} - \frac{{16Q_{a}^{2} }}{{3\pi D_{O}^{2} g}} + 11.334\left( {\frac{{Q_{a}^{2} D_{O}^{2} \sigma_{a} }}{{\rho_{a} g^{2} }}} \right)^{1/3} } \right]$$

**Table 3:** Important properties of porous media in used.

Properties	Filter paper (FP)	Cellulose powder (CP)
Capillary pressure (*P*_*c*_)	0.93 ± 0.14 kPa	4.90 ± 0.58 kPa
Porosity (*ϕ*)	0.6722	0.6529
Contact angle (*θ*)	83.36 ± 0.87 deg	66.77 ± 2.53 deg
Mean pore radius (*r*_*m*_)	14.14 ± 2.67 μm	11.62 ± 2.02 μm
Permeability (*k*)	8.9827 × 10^–12^ m^2^	6.0008 × 10^–12^ m^2^

where *g* is gravitational acceleration constant, *ψ* is Harkins-Brown (H-B) correction factor (see Supplementary Notes: Harkins-Brown correction factor)^47,48^, and *ρ*_*a*_ and *σ*_*a*_ are density and surface tension of alcohol, respectively. By letting the total volume of injected alcohol as *M*_*a*_, the theoretical number of alcohol droplets (*N*_drop_) is defined in Fig. 2A. In addition, the time between the breakups of two consecutive droplets (*T*_drop_) is found as *V*_*a*_/*Q*_*a*_. Because of the reciprocal term *V*_*a*_ between *N*_drop_ and *T*_drop_, they are inversely related. As it will be shown in locomotion analysis section, both *N*_drop_ and *T*_drop_ directly affect the locomotion of the robot. Figure 2C shows the inverse proportional relation of *T*_drop_ and *N*_drop_ more clearly; note that *N*_drop_ was heavily determined by *D*_*O*_. Given minor fabrication error and the fact that the liquids were injected manually, slight differences in the *N*_drop_ values were observed at the same *D*_*O*_. The horizontal lines inside the interquartiles (IQRs) are medians, which were close to the means, and proportional to *D*_*O*_ for both FP and CP. The IQRs show that the use of FP and a *D*_*O*_ = 3.2 mm was associated with very variable *T*_drop_ values in successive tests, caused by the inherently high variability of paper-based microfluidic devices^49^. Given that the experimental relation between *N*_drop_ and *D*_*O*_, the theoretical *T*_drop_ (i.e. the hollow markers in Fig. 2C) shows good agreement with measured *T*_drop_. However, the theoretical *N*_drop_ overestimated a couple of droplets when *D*_*O*_ was 2.2 mm or 3.2 mm (Supplementary Figure S8) due to the variance of *M*_*a*_ during the manual injection.

The time evolution of the droplet counts for each *D*_*O*_ are presented in Fig. 2D–G. Here, the time of the first droplet breakup was used as a reference, and the times to later breakups are depicted. CP was associated with faster breakup than FP as expected due to the higher flow rate (see Fig. 2B), and smaller *D*_*O*_ values were associated with more droplets of smaller volume. The pump produced a maximum of 13 droplets when *D*_*O*_ = 1.0 mm. By considering small mean difference in *T*_drop_ values (see Supplementary Note: Pumping periodicity), the pump was also working in reasonably periodic manner.

### Marangoni flow visualization during propulsion

**Figure 3:**
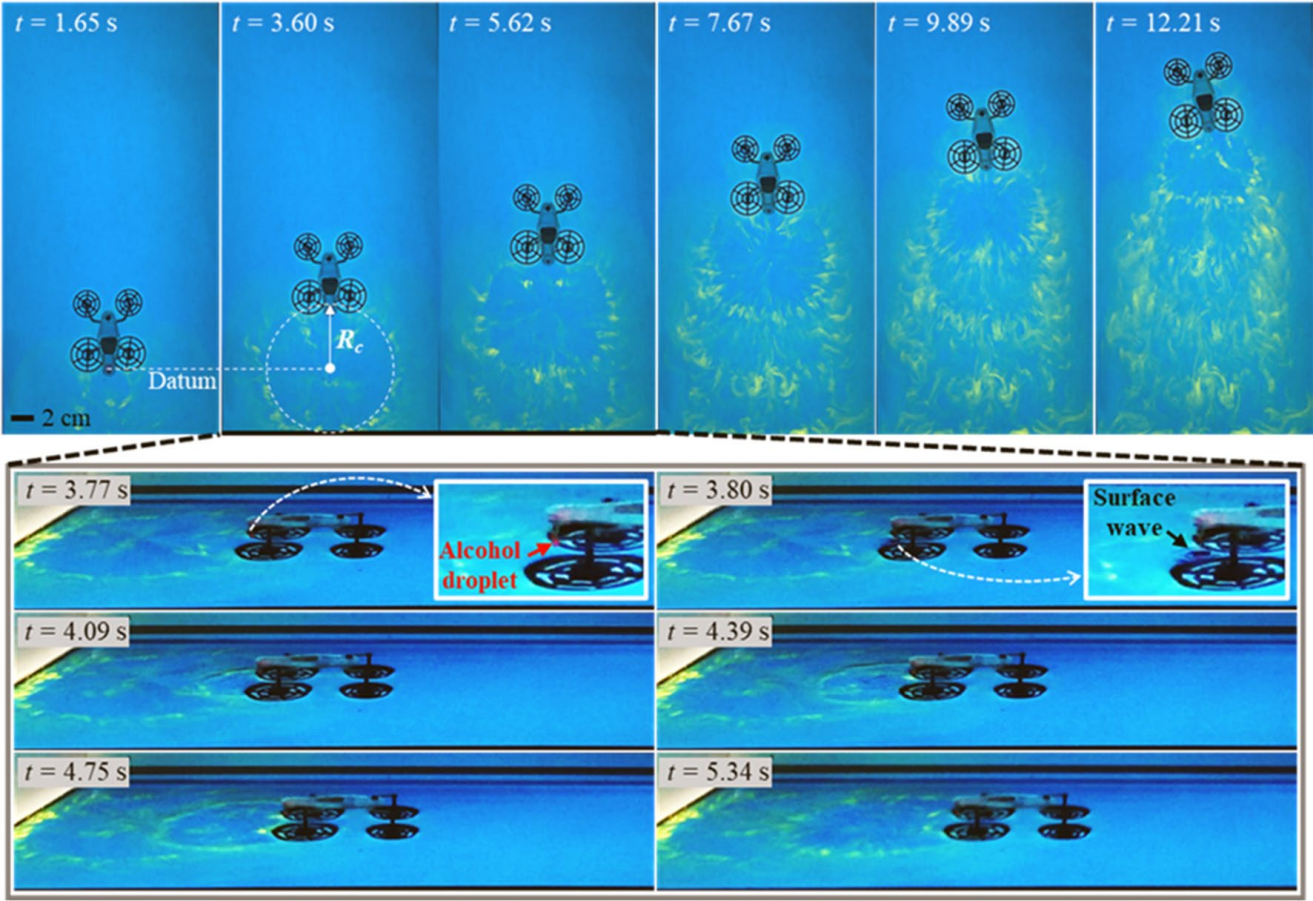
Marangoni flow visualization during propulsion. Top and side views of the robot propelled by the Marangoni effect; flow was visualized using BTB and other chemicals (*R*_*c*_: ring trace radius). CP served as the porous medium. *D*_*O*_ was 3.2 mm and *h*_*f*_ was 9.5 mm; *t* is the time that elapsed after the first alcohol droplet broke from the nozzle.

To visualize induced Marangoni flow, bromothymol blue (BTB), which changes color depending on pH^50^, was used as explained in Supplementary Note: Marangoni Flow Visualization. Alcohol spreading behind the moving robot was revealed in yellow (Fig. 3 and Supplementary Video S5). Each image at the top of Fig. 3 was taken when a droplet was fully spread. Interestingly, the ring trace radius (*R*_*c*_) was almost the same as the travel distance afforded by a single droplet. Also, the center of each ring was the location where the preceding alcohol droplet had coalesced with water.

A side view filmed between 3.77 and 5.34 s shows the radial spreading of a single droplet in more detail, starting with droplet breakup at the nozzle. The radial flow spread was accompanied by many surface waves (see Supplementary Figure S11 for closeups) induced by Marangoni effect, confirming that 3Me1Bu facilitated prolonged motion^34^. However, as an additional chemical was used for visualization, the surface tension difference between the water surface and the propellant was only 57% that of the pure water-and-alcohol case (see Supplementary Note: Marangoni Flow Visualization). The robot actually traveled less distance than on clear water, but interpretation of the trace left on the water surface yielded information on preceding motion.

### Locomotion analysis when *h*_*f*_ = 9.5 mm: complete droplet breakup

**Figure 4:**
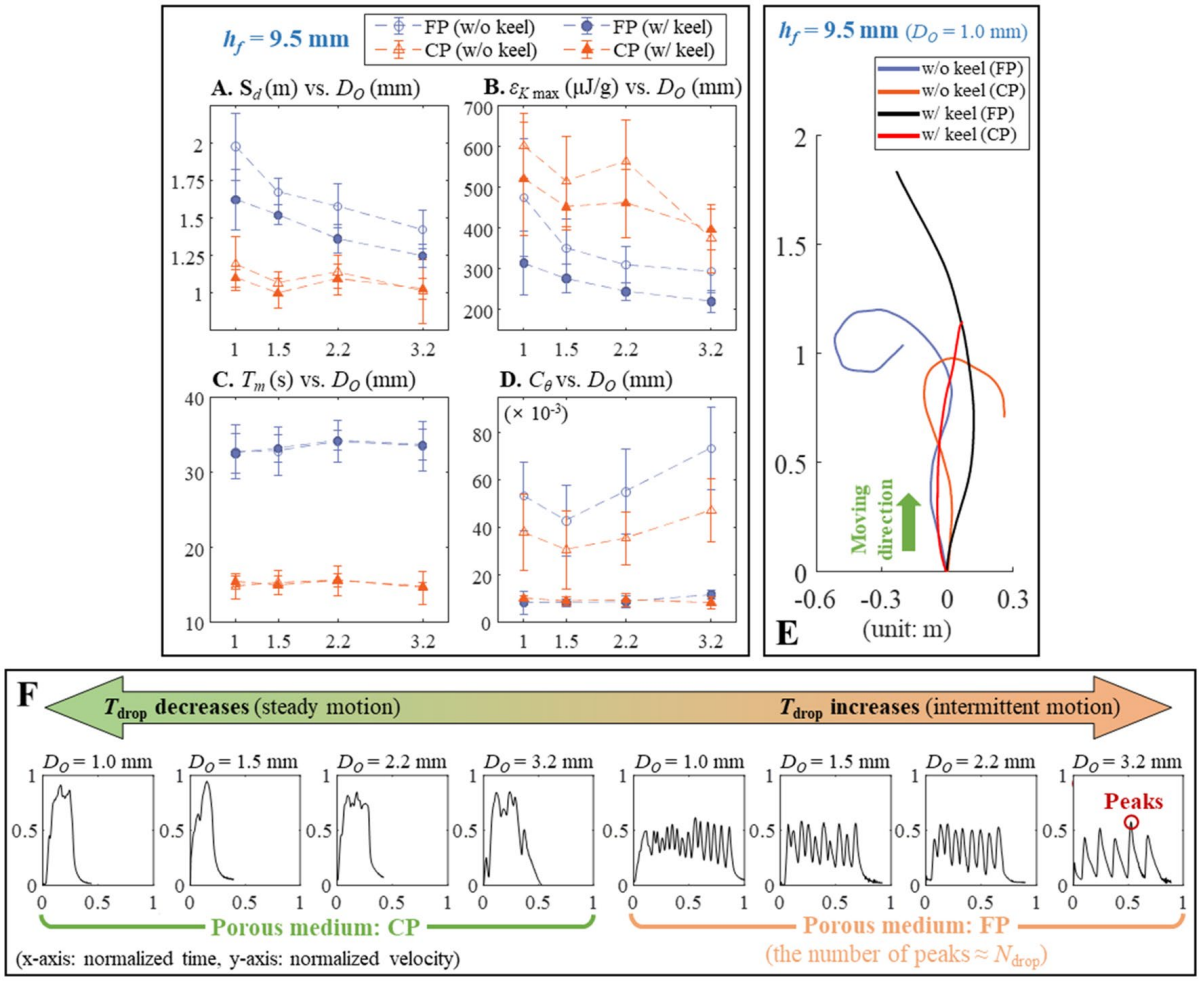
Robot locomotory characteristics at an *h*_*f*_ = 9.5 mm. (**A**) Traveled distance (*S*_*d*_) versus *D*_*O*_. (**B**) Energy density of fuel *ε*_*K*,max_ versus *D*_*O*_. (**C**) Duration of motion *T*_*m*_ versus *D*_*O*_.  (**D**) Moving trajectory characteristic *C*_*θ*_ versus *D*_*O*_ (*n* = 5 for **A**–**D**). (**E**) Moving trajectories of some selected cases. (**F**) The variation of normalized velocity of the robot upon *T*_drop_ change. Note that all the error bars denote standard deviations.

For comprehensive locomotion analysis, we compared traveled distance *S*_*d*_, energy density of fuel *ε*_*K*,max_, duration of motion *T*_*m*_, and trajectory characteristic *C*_*θ*_ in Fig. 4A–D by varying the porous media (either CP or FP), alcohol outlet diameter *D*_*O*_, and keel status while holding the foot height *h*_*f*_ (Fig. 2A) at 9.5 mm. Note that letting *h*_*f*_ = 9.5 mm ensured complete droplet breakup from the nozzle (see Supplementary Figure S12A,C). Thus, *T*_drop_ and *N*_drop_ analyzed in Fig. 2 can directly affect the locomotion on water surface in predictable and intuitive manner. Here, *ε*_*K*,max_ was defined as:

2 $$\varepsilon_{K,\max } = \frac{{m_{{{\text{fuel}}}} }}{{2m_{{{\text{fuel}}}} }}V_{\max }^{2}$$

where *m*_robot_ and *m*_fuel_ are the masses of the robot and injected fuel respectively and *V*_max_ is the maximum propulsion speed^12^; its unit [μJ/g] indicates that it is equivalent to the maximum kinetic energy per unit mass. In the case of *C*_*θ*_, it is dimensionless and quantitatively characterizes the effect of the keel on directional motion, indicating how the heading angle changes during propulsion. Its definition is given below:

3 $$C_{\theta } = \frac{{\Delta T(\theta_{1} + \theta_{2} + \cdots + \theta_{n - 1} )}}{{\pi T_{m} }}$$

where Δ*T* is sampling time, and *θ*_1_ + *θ*_2_ + · · · + *θ*_*n*−1_ (unit: radian) are series of heading angle changes in Δ*T* interval. If the robot proceeded linearly, *C*_*θ*_ was close to zero, and vice versa. Overall, both *S*_*d*_ and *ε*_*K*,max_ were inversely related to *D*_*O*_ (Fig. 4A,B). The use of FP (compared to CP) increased the distance traversed at all *D*_*O*_; and CP use was associated with higher *ε*_*K*,max_ values than FP use. Thus, frequent dispensing of small alcohol droplets (i.e. large *N*_drop_ with short *T*_drop_) was associated with a longer *S*_*d*_ and higher *ε*_*K*,max_ than slow dripping of a few but large droplets. Figure 4C shows that the *T*_*m*_ was predominantly affected by the porous medium chosen; the *T*_*m*_ associated with FP use was always longer than that associated with CP use. Thus, fast water-imbibition into a porous medium (using CP) could boost up *ε*_*K*,max_ at the expense of short *S*_*d*_.

For both porous materials, keel-extrusion reduced both *S*_*d*_ and *ε*_*K*,max_ because of the drag imposed by the keel itself; while the inverse relations with *D*_*O*_ were still maintained. However, *C*_*θ*_ obtained from the keel-extrusion was significantly smaller than those of without keels (Fig. 4D). Thus, the keel afforded directional motion as expected and some typical trajectories are depicted in Fig. 4E. Clearly, the red and black curves in Fig. 4E (i.e., smaller *C*_*θ*_) were less curvy than the blue and orange curves. This characteristic offered by the keel-extrusion was repeatable judging from the associated small standard deviations in Fig. 4D. On the other hand, *C*_*θ*_ obtained from the keel-free footpads were characterized by large standard deviation, which implied unordered and arbitrarily trajectories. The relation between *T*_drop_ and locomotory characteristic is more clearly shown in Fig. 4F. Each subgraph exhibits the normalized velocity of the robot (without keeled footpads) versus normalized time. Noticeably, using a larger nozzle and FP facilitated the robot to move in intermittent manner. Also, the number of velocity peaks was almost the same as *N*_drop_ at the same *D*_*O*_; the inversely proportional relation between *D*_*O*_ and *N*_drop_ in Fig. 2C was responsible for this tendency. On the other hand, using CP with a smaller nozzle facilitated steady locomotion with less velocity fluctuation. Thus, the number of velocity peaks was not meaningfully distinguishable even though *D*_*O*_ was changed. The relation between *T*_drop_ and the resultant behavior was still maintained when keel-extruded footpad was employed. As long as a complete droplet breakup is guaranteed, the pump behavior directly affected the locomotion.

### Locomotion analysis when *h*_*f*_ = 6.5 mm: incomplete droplet breakup

**Figure 5:**
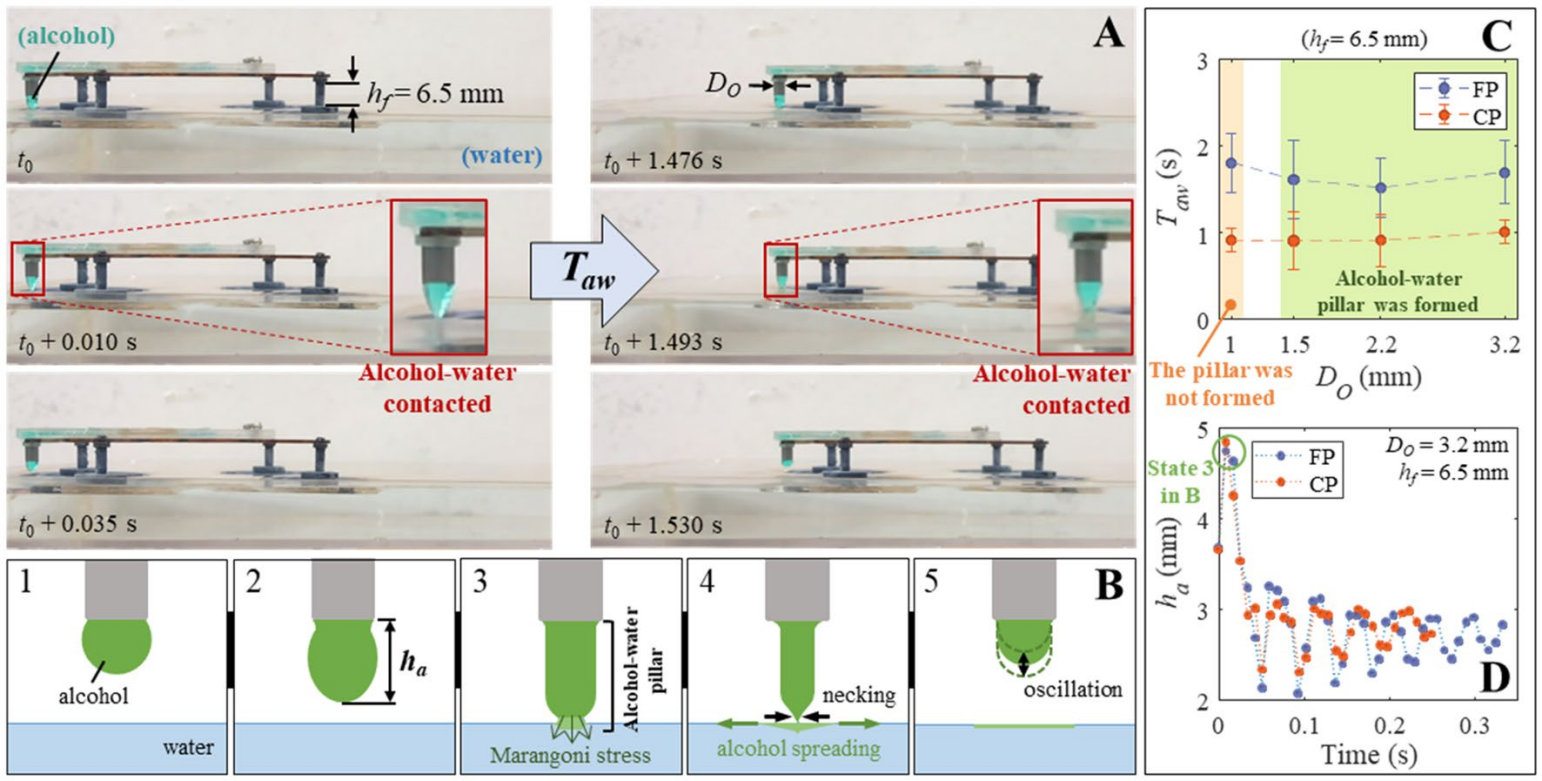
Incomplete droplet breakup at *h*_*f*_ of 6.5 mm forms an alcohol–water pillar. (**A**) Premature alcohol–water contact at an *h*_*f*_ = 6.5 mm (*t*_0_: reference time, *T*_*aw*_: time between two episodes of alcohol–water contact). (**B**) A schematic of alcohol spreading at *h*_*f*_ = 6.5 mm. (**C**) *T*_*aw*_ versus *D*_*O*_ (*n* = 5). (**D**) Oscillation of alcohol column height (*h*_*a*_). Note that all the error bars denote standard deviations.

Reducing *h*_*f*_ = 6.5 mm promoted earlier contact of alcohol to the water surface (Fig. 5A,B) except when *D*_*O*_ = 1.0 mm (see Supplementary Figure S12B,D). Notably, an alcohol–water pillar was momentarily observed (Supplementary Video S6–S7). At the same time, the alcohol spread radially because of the outward Marangoni stress, followed by necking (scenes 3–4 in Fig. 5B)^51,52^. The time between two consecutive alcohol–water contacts (*T*_*aw*_) was noticeably reduced (Fig. 5C), thus to less than the *T*_drop_ in Fig. 2C. Finally, the remaining alcohol dangling at the nozzle began to oscillate, especially when *D*_*O*_ = 3.2 mm (Fig. 5D). The impact of this incomplete droplet breakup had affected the locomotion of the robot.

**Figure 6:**
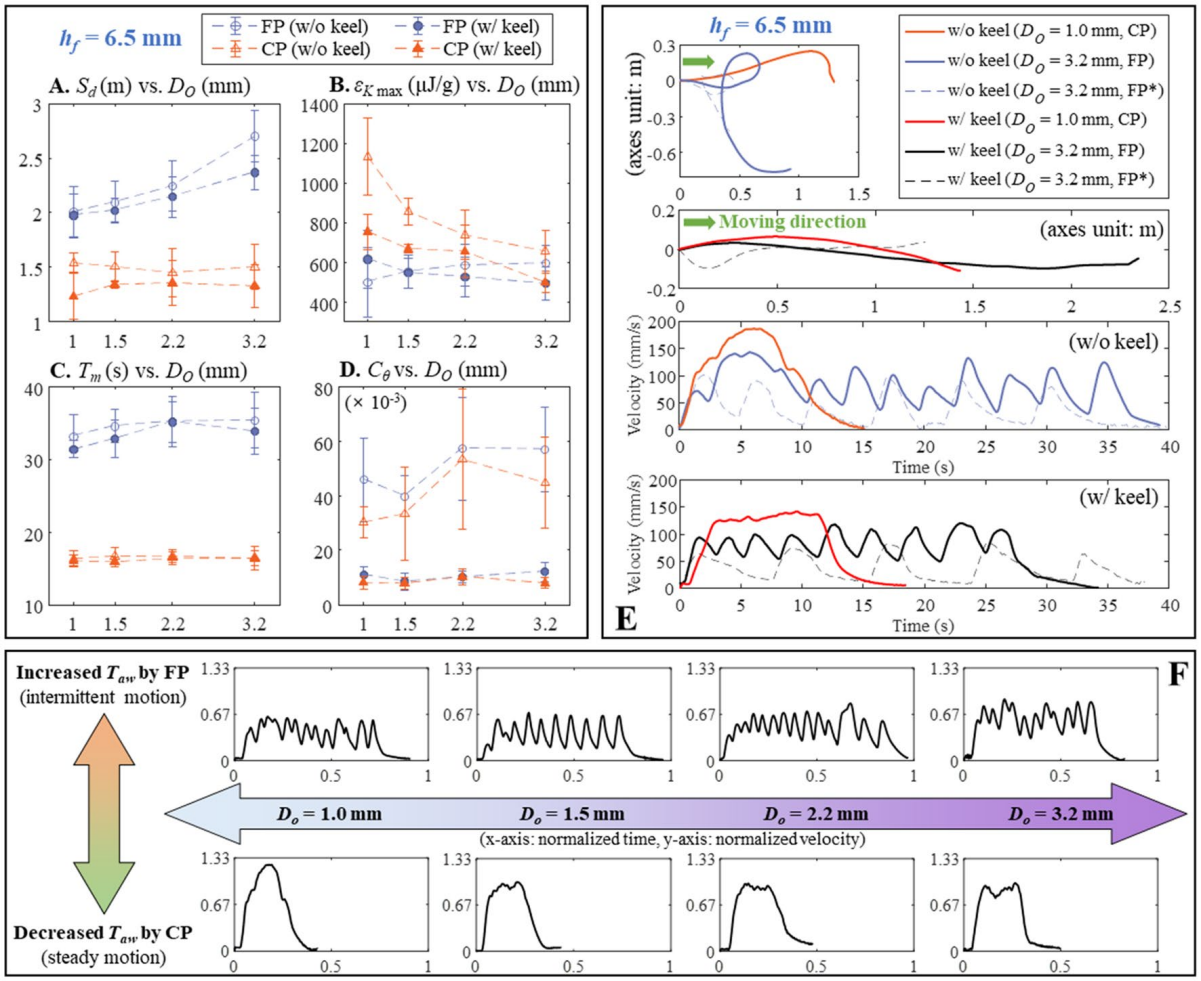
Robot locomotory characteristic at *h*_*f*_ = 6.5 mm. (**A**) Traveled distance (*S*_*d*_) versus *D*_*O*_. (**B**) Energy density of fuel *ε*_*K*, max_ versus *D*_*O*_. (**C**) Duration of motion *T*_*m*_ versus *D*_*O*_. (**D**) Moving trajectory characteristic *C*_*θ*_ versus *D*_*O*_ (*n* = 5 for **A**–**D**). (**E**) Variation of velocity and corresponding moving trajectories of some selected cases. (**F**) The variation of normalized velocity of the robot upon *T*_*aw*_ change; note that Fig. 4F and (**F**) were normalized in the same scale. Note that all the error bars denote standard deviations.

The resultant traveled distance and energy density of fuel exhibited noticeable differences (Fig. 6A,B). Note that it was *D*_*O*_ = 1.0 mm and *D*_*O*_ = 3.2 mm that exhibited maximal *S*_*d*_ for *h*_*f*_ = 9.5 mm and *h*_*f*_ = 6.5 mm, respectively. This implies that optimal *h*_*f*_ for maximal *S*_*d*_ would be different if *D*_*O*_ is changed. However, smaller *D*_*O*_ still facilitated larger *ε*_*K*,max_ when CP was used. For the time duration and directionality of motion (Fig. 6C,D), the overall tendencies were the same at both *h*_*f*_ values. The effect of *h*_*f*_ is also evident in the velocity vs. time graphs (Fig. 6E). The velocity of (*D*_*O*_ = 3.2 mm, FP, *h*_*f*_ = 6.5 mm) case exhibits more peaks and less fluctuation than that for the (*D*_*O*_ = 3.2 mm, FP, *h*_*f*_ = 9.5 mm) case, given the more frequent alcohol–water contacts of the former case. When *h*_*f*_ = 9.5 mm, the velocity increased once more after becoming near zero. Given such rises followed by gradual decreases, the resultant *S*_*d*_ at *h*_*f*_ = 9.5 mm (Fig. 4A) was comparatively small. The impact of *T*_*aw*_ to the velocity of the robot is summarized in Fig. 6F. Using CP again facilitated steady motion, while FP induced noticeable velocity fluctuations. Unlike Fig. 4F, however, the number of velocity peaks obtained from FP were hardly correlated with *D*_*O*_. As *D*_*O*_ had less effect to *T*_*aw*_ (Fig. 5C), significant change in the velocity profile was only induced by porous media. This tendency was maintained when the keel was present except the enhanced directional motion at the expense of velocity. Essentially, the time interval of repetitive alcohol–water contacts and the amounts of alcohol discharged for each contact determine the locomotory behavior. When *h*_*f*_ = 6.5 mm, smaller *T*_*aw*_ than *T*_drop_ had enhanced both *S*_*d*_ and *ε*_*K*,max_. This implies that premature contact between (incompletely grown) alcohol and water surface, and underlying dynamics during the alcohol-to-water coalescence (Fig. 5B) are key aspects for efficient locomotion.

### The relations between target performance and design parameters

Each sets of design parameters were specialized at achieving different locomotory performance (refer Supplementary Video S8). To travel the longest distance, using FP and keel-free-footpad is required. At the same time, nozzle diameter should be small for complete droplet breakup (*D*_*O*_ = 1.0 mm, *h*_*f*_ = 9.5 mm), and large for incomplete droplet breakup (*D*_*O*_ = 3.2 mm, *h*_*f*_ = 6.5 mm). To achieve the fast speed, using CP, keel-free-footpad, and small nozzle are required. To maintain directional motion, using a keel-extruded footpad is sufficient. Whereas using FP is sufficient for prolonged operation of the robot independent to other design parameters. In terms of velocity variation, using CP is recommended for steady locomotion. If possible, using small *D*_*O*_ or letting *h*_*f*_ = 6.5 mm facilitates better steady motion. On the other hand, FP is applicable for intermittent locomotion. If possible, using larger *D*_*O*_ or letting *h*_*f*_ = 9.5 mm is recommended.

### Locomotory characteristics of the proposed robot and others

**Figure 7:**
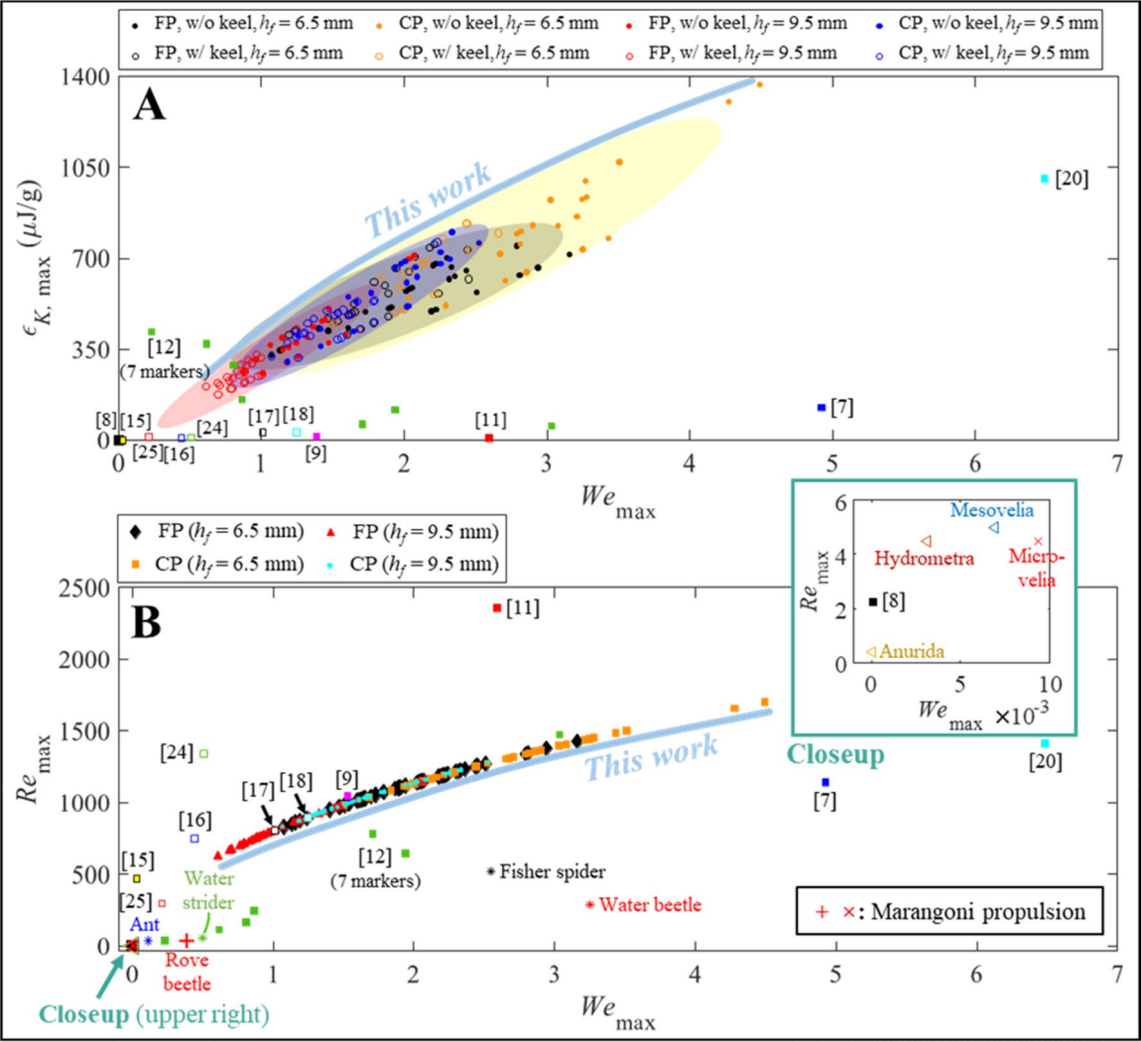
Robot locomotory characteristic using nondimensional numbers. (**A**) *ε*_*K*, max_ versus *We*_max_. Error eclipses include 95% confidence intervals. Identical coloration of markers and eclipses indicate the use of identical porous media and *h*_*f*_ values. (**B**) *Re*_max_ versus *We*_max_. Biological data were obtained from^2, 53–57^, and no distinguish was made between keeled and pristine footpads.

To further compare the locomotory characteristics with other systems and some aquatic arthropods, *ε*_*K*,max_, maximum Weber number (*We*_max_), and maximum Reynolds number (*Re*_max_) were considered. Here, *We*_max_ and *Re*_max_ were dimensionless and defined as below:

4 $$We_{\max } = \frac{{\rho_{w} w_{f} V_{\max }^{2} }}{{\sigma_{w} }},\quad Re_{\max } = \frac{{V_{\max } w_{f} }}{{\nu_{w} }}$$

where *w*_*f*_ was foot width, and *ν*_*w*_ was kinematic viscosity of water^1^. *We*_max_ and *Re*_max_ indicate the importance of inertial force compared to surface tension and viscous force, respectively. Depending on *V*_max_ of the robot, its *We*_max_ was varied from 0.5 to 4.5 (Fig. 7), thus within the ranges of many previous works. In particular, the *ε*_*K*,max_ of the (CP, w/o keel, *h*_*f*_ = 6.5 mm) cases were higher than those in any previous works; the highest *ε*_*K*,max_ in our work was 1368 μJ/g. Note that specific parameters used to depict Fig. 7 are summarized in Supplementary Table S3. In the case of *Re*_max_, its range was also similar to that of previous systems. However, *Re*_max_ of this work was far higher than that of aquatic arthropods. Here, only the red cross and plus markers denoted data originated from Marangoni propulsion; while other markers were obtained from other modes of locomotion for comparison^2, 53–57^. This implies that the robot behavior was dominated by inertia due to its larger body size than arthropods.

## Discussion

Capillarity is one of the fundamental physical phenomena that enables autonomous fluid flow without an external assistance. This study demonstrates how capillary pressure can provide mobility to a robot on water surface. Inspired by the Marangoni propulsion of aquatic insects, a new battery-less self-propulsion robot was developed, which utilized a magnetically triggerable, water-imbibition powered microfluidic pump for the first time. Owing to utilization of Marangoni effect, no electrically driven actuator, or other forms of mechanically moveable parts are required to generate thrust. While previous Marangoni propulsion systems had difficulties in controlling the propellant-transfer to water surface, the dripping behaviors of propellant are mechanically tunable in this work. Our method no longer required a vertically-aligned vessel to generate fuel droplets unlike previous systems^15–18^. Thus, the proposed system could avoid any accompanying issue such as the submergence caused by the unequally distributed load from a fully loaded fuel vessel. The trigger mechanism of the pump employed a novel microfluidic structure, which was not tried in the previous methods^16–18,23,27^. The proposed trigger is electric power free, instantaneous, independent to water surface condition, and free from an external equipment. The pump performance was also comprehensively analyzed. It was shown that the locomotion of the robot can be related with the predicted pump characteristic if the complete droplet breakup is ensured. On the other hand, approaching the nozzle tip closer to water surface increased the complexity of the locomotory behavior. More specifically, the locomotory characteristics were significantly different depending on four parameters: namely, porous materials (FP or CP), keel-extrusion, nozzle diameter (*D*_*O*_), and footpad height (*h*_*f*_ = 6.5 or 9.5 mm). Overall, using CP was characterized by fast propulsion speed in steady manner but with short duration because of the fast water-imbibition rate than FP (refer Fig. 2). Adsorbing the same amount of water with a fast flow rate inevitably reduced the operational time of the pump. Thus, using FP was associated with prolonged propulsion than CP because of its smaller flow rate than CP. In the case of keel-extrusion, it certainly helped the robot to maintain directional motion independent to other parameters (refer Figs. 4D, 6D). This was possible due to the asymmetric drag force generated by the keel, which already explained in Supplementary Note: Drag asymmetry. Whereas the effects of *D*_*O*_ and *h*_*f*_ were tightly coupled as a small *h*_*f*_ facilitated premature contact of a propellant droplet to the water surface. When *h*_*f*_ = 9.5 mm (i.e., complete droplet breakup), increasing *D*_*O*_ reduced both the traveled distance (*S*_*d*_) and maximal propulsion speed (or *ε*_*K*, max_) due to the relatively long *T*_drop_ at large *D*_*O*_ (refer Fig. 2C). An overly long *T*_drop_ could not provide a propulsive force while the robot was still propelled by a prior propellant. Intermittent propulsion after when the velocity of the robot was reduced nearly zero had a disadvantage in achieving a longer traveled distance with fast propulsion speed. On the other hand, reducing *h*_*f*_ down to 6.5 mm complicated all the kinematic result as reported in Fig. 6. But, both *S*_*d*_ and *ε*_*K*, max_ was improved owing to the frequent dispensing of propellant (refer Fig. 5C) than the dispensing rate associate with *h*_*f*_ = 9.5 mm (i.e. *T*_drop_ in Fig. 2C).

Changing the porous material effectively changed the flow rate of the pump, and similar result can also be obtained by varying the microchannel-porous medium heights (*H*). As already addressed in equations S10 and S16 (Supplementary Note: Flow rate model), *H* was proportional to the flow rate. Thus, increasing *H* would have similar results of switching the porous medium from FP to CP: reduced *T*_drop_, *S*_*d*_, and *T*_*m*_, but increased *ε*_*K* max_. On the other hand, decreasing of *H* will exhibit the opposite locomotory behaviors.

The proposed system, however, exhibited noticeable performance-variability caused by multi-layered filter papers and manually packed cellulose powders. Thus, a tailored porous medium should be fabricated in desired size and shape for consistent performance. Also, the density of microchannel should be increased (to hold more fuel) for a given volume of the pump to increase the limited operation time. In addition, measuring and modeling of the time-varying Marangoni propulsion force in our system is still remained as a great challenge^58^. This work is still a foundational study, which integrated a microfluidic device to a liquid interface traveling system, and future investigation is required for its potential applications. For example, the proposed system can be used to environmental remediation of liquid interface^22^. Besides, the functionality of on-board microfluidic device can be improved by integrating microfluidic digital logic^59^, or a passive microfluidic mixer^60^ to induce on-site chemical reactions.

## Methods

### Preparation of porous media

Off-the-shelf filter papers (see Supplementary Table S1) and cellulose powder (Daejung Chemicals) were used. Filter papers were cut with a CO_2_ laser machine. The particle retention and basis weight specified in Supplementary Table S1 were obtained from the manufactures’ datasheet. While the total thickness was measured using a digital caliper in 0.01 mm resolution. The particle size distribution of the cellulose powder was obtained from a particle size analyzer (Mastersizer 3000. Malvern).

### Fabrication of the proposed robot

An exploded view of the robot is given in Supplementary Figure S13. The pump was composed of two layers made of polydimethylsiloxane elastomer (Sylgard 184, Dow-corning), and both layers were molded and cured in an oven for 2 h at 60 °C after pouring liquid state elastomer into 3D printed molds. These two layers were bonded each other using a corona plasma treater (Corona SB, BlackHole Lab) followed by bonding a neodymium magnet to the bottom layer with an adhesive (Loctite 401, Henkel). A SUS nozzle was also tightly fitted into the outlet. After that, a laser-cut 0.8 mm thick polycarbonate body frame was bonded with the pump using an adhesive layer (468MP, 3 M). The circular footpads were made of 3D printed acrylonitrile butadiene styrene. Whereas a 0.15 mm thick epoxy-impregnated glass fiber plate was for the keel. The footpads were all spray coated with water-repelling material (NeverWet, RUST-OLEUM) for hydrophobicity. Before injecting water and alcohol to the microchannels, a porous medium is filled inside the designated chamber. The porous medium was gently pressed with a laser-cut polycarbonate cover followed by sealing with an adhesive tape for airtightness.

### Measurement of capillary parameters

To theoretically calculate the flow rate of water-imbibition, capillary pressure (*P*_*c*_), porosity (*ϕ*), contact angle (*θ*) between water and porous medium, and mean pore radius (*r*_*m*_) were required. Here, *P*_*c*_ was obtained by measuring the maximum blockage pressure at the terminal of a porous medium while water was being imbibed into it (Supplementary Note: Capillary pressure measurement). The porosities of filter papers and cellulose powder were found by using mercury porosimetry (Supplementary Note: Porosity measurement). Lastly, *θ* and *r*_*m*_ were found according to the previous method^61^; they were parameterized to a nonlinear fitting problem of water adsorption mass versus time (Supplementary Note: Contact angle and mean pore radius).

### Filming and measurement of dripping interval, flow rates, and alcohol droplets

All the time intervals in Fig. 2 were obtained by filming the outlet nozzle at 30 fps (frame-per-second) and inspecting the moment of droplet breakup frame-by-frame. Here, the pump was fixed to a stationary platform, and each case was repeated 15 times. To measure the flow rates in Supplementary Figure S6A, a transparent film printed with calibrations along the microchannel was aligned on the pump. Then, the entire pump operation was filmed (FP: 60 fps, CP: 120 fps). We also employed another camera directing to the outlet nozzle to identify the moments of droplet breakup. Flow rate was calculated by multiplying the cross-section area of the microchannel (0.75 mm^2^) with the speed of water or alcohol moving inside. As we already knew all the distances between two calibrations, the moving speed of the liquids for each unit distance could be easily known. The average flow rates in Fig. 2B and Supplementary Figure S6B were obtained from the series of flow rate vs. time graphs. The alcohol–water pillars shown in Fig. 5A were recorded by filming the robot moving on a water filled petri dish (size: 24 × 24 × 2.5 cm^3^) at 120 fps. After that, *T*_*aw*_ was obtained by measuring the time interval of the contact between alcohol and water had sequentially occurred. In the case of Supplementary Figure S12, the same water-filled petri dish was used; however, the robot was set to immovable (by placing heavy weights) to get clear images (*h*_*f*_ = 9.5 mm: 60 fps, *h*_*f*_ = 6.5 mm [*D*_*O*_ = 2.2, 3.2 mm, FP]: 60 fps, *h*_*f*_ = 6.5 mm [*D*_*O*_ = 1.0, 1.5 mm]: 240 fps, otherwise: 120 fps). The oscillation of alcohol height *h*_*a*_ was measured by using ImageJ.

### Marangoni flow visualization

To demonstrate intuitive Marangoni flow visualization during the propulsion of the robot, bromothymol blue (BTB) was utilized, which was changed into yellow and blue color when pH was acidic and alkaline, respectively. Thus, we adjusted the pH of water inside a 25 × 75 cm^2^ sized water tank to 7.6 (weak alkaline); while the pH of alcohol fuel was set to 1.3 (strong acidic). More detail procedures were fully described in Supplementary Note: Marangoni Flow Visualization.

### Force measurement of the footpads

Maximal support force was measured by monitoring the sudden drop of reaction force while a footpad was slowly approaching toward water surface until the integrity of water surface was broken (Supplementary Note: Maximal supporting force). Whereas, dragging a keel-extruded footpad at constant speed inside a water tank could calculate the drag asymmetry in parallel flow direction and normal direction (Supplementary Note: Drag asymmetry).

### Motion measurement on water surface

The locomotion of the robot was measured with eleven motion capture cameras (Prime 13, Optitrack) placed around a 3.5 × 2.1 m^2^ sized water pool. A marker was attached to the robot for tracking its moving trajectory at 120 Hz. If certain data was partly lost during the measurement, cubic spline was performed to fill the gap. Also, a low pass filter, which was pre-implemented in Motive software (Optitrack), was applied to the position data at 4 Hz of cut-off frequency to remove spikes. The instantaneous velocity of the robot was obtained by dividing 1/120 s (i.e. the inverse of data acquisition rate) from the moving distance. This was then post-processed with butterworth filter at 3 Hz cut-off frequency using Matlab (Mathworks). All the locomotion analysis in Figs. 4, 6, and 7 were acquired in the same manner and repeated 5 times.

## Supplementary Information

Supplementary Information 1.

Supplementary Video S1.

Supplementary Video S2.

Supplementary Video S3.

Supplementary Video S4.

Supplementary Video S5.

Supplementary Video S6.

Supplementary Video S7.

Supplementary Video S8.
