# Supplementary material for: Marangoni effect inspired robotic self-propulsion over a water surface using a flow-imbibition-powered microfluidic pump
Source: Sci Rep. 2021 Sep 1;11:17469. doi: 10.1038/s41598-021-96553-8 (PMC8410760; doi:10.1038/s41598-021-96553-8)
Supplement: Supplementary file 1 — Supplementary Information 1. [file 41598_2021_96553_MOESM1_ESM.docx]

Supplementary Notes

**Note: Maximal supporting force**

The circular footpad shown in Fig. 1B was useful at Marangoni propulsion as previously demonstrated in^16–18^*^,^* ^33^. Here, a 0.15 mm thick keel was extruded 15 mm from the bottom surface to demonstrate the directional motion of the robot. The whole surfaces of the footpad were spray coated with water-repelling material (NeverWet, RUST-OLEUM) for hydrophobicity. To measure the maximal support force (*F_s_*_,max_) of the footpad, an experimental setup illustrated in Supplementary Fig. S1(A) was prepared. A force/torque sensor (ATI Nano 17) was installed between a footpad and a moving stage to measure the reaction force while the footpad was slowly being contacted with water surface at the speed of 1 mm/s.

The variation of reaction force (*F_s_*) during the experiment typically followed the graph shown in Supplementary Fig. S1(B). This result was obtained at 100 Hz of acquisition rate, and no post process (e.g. filtering) was performed. When the keel was being contacted with the water surface at *t*_1_, its *F_s_* was not noticeably changed. However, *F_s_* was increasing at *t*_2_ as the footpad was making a contact with water until the integrity of its surface was lost at *t*_3_. Here, *F_s_*_,max_ was also reached at *t*_3_ just before the breakage of the water surface followed by sudden drop of *F_s_*. The footpad was then completely submerged under the water surface at *t*_4_. At the final stage, the footpad was retraced from the water surface, and *F_s_* was restored to zero at *t*_5_.

The *F_s_*_,max_ of four different types of footpads were measured five times for each case, and the result is given in Supplementary Fig. S1(C). As expected, *F_s_*_,max_ of the rear footpads were greater than the frontal footpad owing to its long perimeter. Also, it was shown that additional *F_s_*_,max_ provided by the keel was comparatively small due to its thin layer. Since the proposed robot had two frontal and rear footpads, respectively, the total *F_s_*_,max_ would be 188 mN if the keel was not employed. While it was increased to 194 mN when all the footpads were equipped with the keels. As the total mass of the robot with and without the keel was 6.91 g and 6.15 g, respectively, the *F_s_*_,max_ of the four footpads was almost three times greater than the robot’s own weight. Because of the repetitive surface tension reduction and gradual degradation of the water-repellant coating by alcohol droplets, that amount of *F_s_*_,max_ was enough to avoid submergence.

**Note: Drag asymmetry**

A keel-extruded footpad was used to demonstrate the directional motion of the robot. To experimentally identify the drag force (*F_D_*) difference when water is flown parallel along or normal to the keel, an experimental setup depicted in Supplementary Fig. S2(A) was prepared. The same moving stage and force/torque sensor used in Supplementary Note (Maximal supporting force) were used again; however, the footpad was horizontally towed at this time. Since the resolution of the force/torque sensor was 1 mN, which was not sufficient to measure *F_D_*, the applied torque divided by the length of a connecting rod was considered as *F_D_*. To select one representative towing speed of the footpad, the average moving speeds of the robot obtained from entire experiments conducted in this work were considered as shown in Supplementary Fig. S2(B). Since 70 mm/s was the mean speed from that distribution, it was chosen as the towing speed. A footpad was towed in water-filled container along 280 mm in each trial, and data acquisition rate was 100 Hz. Here, time averaged drag force (*F_D_*_,avg_) defined below was calculated:

$F_{D,\text{avg}}=\frac{\int_{0}^{T} F_{D}(t)dt}{T}$ (S1)

where *T* was measurement time. The *F_D_*_,avg_ of a keel-extruded footpad in parallel and normal flow directions were independently measured where the parallel direction was identical to the intended moving direction of the robot. On the other hand, flow direction was not considered for a pristine (i.e. w/o keel) footpad. Each case was measured five times, and the results are compared in Supplementary Fig. S2(C). Overall, the drag force in normal direction was 5.3 times greater than the parallel direction in both frontal and rear keel-extruded footpads. Owing to this drag asymmetry, the robot was forced to move in forward direction by suppressing any side-way perturbation. The *F_D_*_,avg_ of the pristine footpad was similar to that of the keel-extruded footpad aligned in parallel direction. As expected, no drag asymmetry was observed from the pristine footpads.

**Note: Porous media selection**

We prefixed the geometry of a porous medium as a trapezoid whose width is gradually decreased along the flow direction to facilitate fast flow speed^35^. In addition, all the four corners were rounded to avoid air traps during flow-imbibition.

The volumetric flow rates of alcohol propellant while not generating any droplet were compared among 10 porous media. Nine of them were off-the-shelf filter papers made of cellulose, and their specifications are summarized in Supplementary Table S1. The thickness of a single filter paper was typically ranged between 0.1 mm and 0.2 mm except FC 1113. As the height of the microchannel inside the pump was 0.75 mm, multiple filter papers need to be stacked to maximize the volume of imbibed water. Thus, the number of filter layers was adjusted to set its total thickness close to 0.75 mm. Unlike the finite number of stacked filters, using granular material can eliminate flow resistive effect in minute gaps between interfaced filter papers. Thus, cellulose powder (CP) was also considered as a porous medium, and its particle size was distributed from 10 μm to 200 μm, while the mean particle size was 59.8 μm.

To avoid any effect caused by droplet formation while comparing flow rates, only 30 μL of alcohol was injected to the pump; this amount could fill about the quarter of the entire microchannel reserved for alcohol-injection. At first, average flow speed was obtained by dividing the traveled distance (*L_a_*) of the injected alcohol from state 1 to state 2 shown in Supplementary Fig. S3(A) with elapsed time *t*_elp_. Then, its flow rate was obtained by multiplying the cross-section area of the microchannel *A* to *L_a_*/*t*_elp_, and the result is shown in Supplementary Fig. S3(B). Note that the effect of extrusion at the flow-imbibition inlet was also compared for the filter papers, which exhibited noticeable impact in successful rate of the magnetic clamping. The display orders of the porous media (including CP) in Supplementary Fig. S3(B) were based on their flow rates of non-extruded types.

Overall, no strict correlation was found between the resultant flow rates by filter papers and their specifications (e.g. particle retention, basis weight), which provided by the manufactures. This was caused by the discrepancy of the filter paper layers due to the 0.75 mm height constraint. Under this restriction, both non-extruded MN 617 and WM 4 exhibited fast flow rates. Meanwhile, the flow rate of CP was as high as MN 617 or WM 4.

In the case of extruded filter papers, all their flow rates were decreased about two-fold compared with non-extruded filter papers. Also, the decreasing order the flow rates (from MN 617 to FC 1113) was quite similar to that of non-extruded ones. The cause of such flow rate reduction was obviously the extrusion itself whose both width and length were 0.75 mm identically (i.e. *L*_1_ = *W*_1_ = 0.75 mm in Supplementary Fig. S3(B)). The fluid transport time *T_f_* through a two-sectioned rectangular porous medium can be found as:

$T_{f}=\frac{\mu V}{k\Delta PH}\left(\frac{L_{1}}{W_{1}}+\frac{L_{2}}{W_{2}}\right)$ (S2)

where *μ* is fluid viscosity, *k* is permeability, Δ*P* is lengthwise pressure difference, *V* and *H* is the volume and thickness of a porous medium, respectively^36^. Although the shape of the filter paper shown in Supplementary Fig. S3(B) was not a rectangle, we let *W*_2_ as 12 mm, which was the width of the trapezoidal part of the filter paper at its geometric center; while the exact value of *L*_2_ was 14 mm. If we assumed the *V* and Δ*P* of extruded and non-extruded filters be the same due to the small size of the extrusion itself, then, *T* of an extruded filter was almost two times larger than a non-extruded filter. Note that *L*_1_ and *W*_1_ are absent in the case of non-extruded filter. Thus, introducing such a small extrusion can cause noticeable change in flow rates.

Overall, both MN 617 filter paper and CP exhibited fast flow rates compared with other porous media. After successfully triggering the pump, however, the flow imbibition was discontinued at times, which eventually ceased the pump operation. Therefore, we also compared the successful rates of the magnetic clamping when MN 617 and CP were used. Although introducing a small extrusion reduced its resultant flow rate about two folds, this also greatly improved its successful rate. In the case of MN 617, the successful rate of non-extruded type was increased from 0.44 to 0.96 by introducing an extrusion during 25 times of trials. Meanwhile, CP also exhibited successful rate of 0.84 in the same condition. In summary, the flow rate of MN 617 was the highest among all the extruded filter papers considered, while CP exhibited even higher flow rate. On the other hand, the successful rate of extruded MN 617 was higher than CP. By considering these results, both extruded MN 617 and CP were utilized as porous media.

**Note: Capillary pressure measurement**

It is widely known that capillary pressure (*P_c_*) is responsible for driving the liquid imbibition into a porous medium. In other words, *P_c_* is pressure difference across an interface formed by the frontal line of liquid-filled porous medium and the remaining air-filled (i.e. unwetted) part, and it can be found as:

$P_{c}=\frac{2\gamma_{w}\cos\theta}{r_{m}}$ (S3)

where *γ_w_* is surface tension of water, *θ* is contact angle between water and a porous medium, and *r_m_* is its mean pore radius^37^. Note that unlike *γ_w_*, both *θ* and *r_m_* were unknown. Thus, they were experimentally found in Supplementary Note (Contact angle and mean pore radius). In this supplementary note, only the resultant *P_c_* was obtained by measuring the maximum gauge pressure at the terminal of a porous medium as shown in Supplementary Fig. S4.

A modified microfluidic chip was prepared by increasing the length of the water channel while eliminating the alcohol channel. The fabrication process of this chip was the same as the proposed pump. Here, the terminal of a porous medium was connected to a pressure sensor (XGZP6847, CFSensor), and it was completely sealed from ambient environment to measure gauge pressure. After filling water and a porous medium, the same magnetic clamping was employed to trigger the pump at *t*_1_ (refer Supplementary Fig. S4). As expected, the gauge pressure was gradually increased during the water-imbibition, which was driven by *P_c_*. If the gauge pressure at the terminal is greater than *P_c_*, then the frontal line of unabsorbed water inside the channel was disconnected from the entrance of the porous medium at *t*_3_. Immediately after the disconnection, the remaining water was repelled toward the water injection hole. In the meantime, the gauge pressure was then gradually decreased at *t*_4_. In this study, we let the maximum gauge pressure as *P_c_*, and repeated the same experiment eight times for each of porous medium. The *P_c_* of FP and CP was 0.9333 ± 0.1422 kPa and 4.8964 ± 0.5847 kPa, respectively. This result is revisited in Supplementary Note (Contact angle and mean pore radius) to validate the measurement of *θ* and *r_m_*.

**Note: Porosity measurement**

Porosity (*ϕ*) is a dimensionless number, which is ranged between 0 and 1, to indicate the volumetric fraction of void in a porous medium. For example, a solid material that absolutely free from any avoid has *ϕ* = 0. To identify the porosity of FP and CP, a mercury porosimetry (AutoPore 9520, Micromeritics) was used. Experimentally found porosity of FP and CP was 0.6722 and 0.6529, respectively.

**Note: Contact angle and mean pore radius**

In this section, *r_m_* and *θ* are obtained by measuring the weight change of a porous medium over time during water imbibition^61^. The liquid uptake per unit area (*m*) of a porous medium is described by the following differential equation:

$\frac{dm}{dt}=b\left(\frac{m_{\infty}}{m}-1\right)$ (S4)

where *t* is time, *b* and *m*_∞_ are model parameters defined as below:

 (S5)

where *ϕ* is porosity found in Supplementary Note (Porosity measurement), *ρ_w_* is density, *ν_w_* is kinematic viscosity, *σ_w_* is surface tension of water, and *g* is gravitational acceleration constant. Here, the solution of (S4) is known as:

$t=-\frac{1}{b}\left(m+m_{\infty}\ln\left(1-\frac{m}{m_{\infty}}\right)\right)$ (S6)

By experimentally measuring *m* over time *t*, both *b* and *m*_∞_ can be obtained by using nonlinear regression, and Levenberg Marquardt method was chosen^61^. After that, *r_m_* and *θ* can be determined from (S5). The schematic of a measuring apparatus to obtain *m* is shown in Supplementary Fig. S5(A). The measurement was made down to fourth places of decimals in gram with an analytical balance (DVG214C, OHAUS). This level of precision was necessary to capture the subtle changes of weight during the imbibition; however, the effect of evaporation become non negligible as well. Thus, a petri dish cover and a balance cover were installed to reduce the evaporation during experiment. In addition, average evaporated weight per second (*e*_avg_) was considered as follows:

$m(t)=\frac{w(0)-w(t)+e_{\text{avg}}t}{A_{\text{pm}}} (t=1,2,\cdots,T)$ (S7)

where *w*(*t*) is measured weight by the balance, *A*_pm_ is the cross-sectional area of a porous medium perpendicular to the water-imbibition direction, and *T* is total measurement time. Note that the measurement was made in 1 second of interval, which was the fastest data acquisition rate provided by the balance. It was also important to use a wide petri dish to minimize the surface height change caused by the water-imbibition, thereby maintaining the contact between the water surface and the sample for as long as possible. Typical measurement results obtained from the FP sample and CP sample are given in Supplementary Fig. S5(B) including the solution given by (S5) after performing the nonlinear regression. By repeating the same experiment five times for each porous medium, *θ* and *r_m_* of FP was found as 83.86° ± 0.87^°^ and 14.14 μm ± 2.67 μm, respectively. In the case of CP, they were 66.77° ± 2.53° and 11.62 μm ± 2.02 μm. The corresponding capillary pressure *P_c_* calculated from (S3) was 1.1133 ± 0.0808 kPa and 5.0121 ± 0.6578 kPa for FP and CP, respectively. These calculated results were also close to the measurements given in Supplementary Note (Capillary pressure measurement), which were fell within 95% of confidence interval.

**Note: Average flow rates**

Typical measurement of flow rate when *D_O_* = 1.0 mm is presented in Supplementary Fig. S6(A). Note that the water channel is longer than the alcohol channel to ensure complete dispensing of alcohol. Thus, the last droplet often broke from the nozzle after the alcohol flow ’visible’ from above had entered the nozzle; the last droplet breakup of Supplementary Fig. S6(A) using FP is one such example. The initially high flow rate of water fell rapidly to attain a quasi-steady state about 2 s after clamping; the alcohol flow rate was delayed by about 1 s. Notably, the alcohol flow rate decreased momentarily when an alcohol droplet broke from the nozzle, caused by the sudden evolution of a droplet interface at that moment^38^; this phenomenon was evident at the rest of *D_O_* values.

Despite the fluctuation of alcohol flow rate, both flow rates of water and alcohol were still bounded. Thus, the average flow rates shown in Supplementary Fig. S6(B) were derived with exclusion of only the initial 1 s periods; all tests were performed five times. As expected, CP (compared to FP) use was associated with larger *Q_w_* and *Q_a_* values for all *D_O_*. As the variations in *Q_w_* and *Q_a_* at different *D_O_* values were quite small, the average flow rates given in Fig. 2B were obtained by combining all the measurement data (regardless of *D_O_*) to validate the modeling accuracy of water imbibition.

**Note: Flow rate models**

Theoretical calculation of flow rate enables us to predict the dripping behavior of the proposed pump. To calculate *Q_w_*, two different imbibition models shown in Supplementary Fig. S7(A-B) were considered; one is assuming circular expansion (CE) of water imbibition, while the other assumed elliptical expansion (EE). Both *W*_1_ and *L*_1_ were 0.75 mm in this study. It was necessary to identify the capillary pressure (*P_c_*) that drove water imbibition, as well as porosity (*ϕ*), contact angle (*θ*), and mean pore radius (*r_m_*) of both FP and CP. These were experimentally determined from Supplementary Notes (Capillary pressure measurement, Porosity measurement, Contact angle and mean pore radius) and summarized in Table 3. Another important property of a porous medium is permeability (*k*). It is independent to the nature of fluid; however, *k* is determined by the geometry of a porous medium. Fortunately, various deterministic models to calculate *k* using *r_m_* and *ϕ* were available^39–42^. In this study, Kuwabara (KB) and Carman-Kozeny (CK) permeability models were considered owing to their accuracy, which described as below:

 (S8)

where *K* is an empirical parameter. In the case of circular expansion (CE) model, the evolution of fluid front *R_f_* (*t*) during imbibition is modeled as a semicircle shown in Supplementary Fig. S7(A). Then, its flow rate *Q*(*t*) and capillary pressure *P_c_* are modeled as below^37^:

 (S9)

Here, equating and rearranging the two equations in (S9) resulted in the flow rate associated with CE model (*Q*_CE_(*t*)) below:

$Q_{CE}(t)=\frac{kHP_{c}}{\mu\left(\frac{L_{1}}{W_{1}}+\frac{1}{\pi}\ln\left(\frac{2R_{f}(t)}{W_{1}}\right)\right)}$ (S10)

where *μ_w_* is dynamic viscosity of water, *t* is time, and *H* = 0.75 mm is the thickness of a porous medium. It is important to calculate *R_f_* = *R_f_* (*t*) to evaluate *Q*_CE_(*t*), and therefore the radial fluid flow model was utilized^43^. A fan-shaped porous medium with central angle of *ω* is considered where *R*(*t*) is the radius of wetted area at time *t* and *R*_0_ is that of fluid source. Then, its fluid flow is described by:

 (S11)

Since *ω* = *π* in our case, we let *R*_0_ as 0.5*W*_1_; in other words, 2*R*_0_ was considered as the width of the fluid inlet whose size was *W*_1_. By letting *R* = *R_f_* (*t*), the solution of (S11) can be used to obtain *Q*_CE_(*t*). After multiplying the both sides of (S11) with 2*e*^−1^ and some rearrangements, (S11) was reformulated as:

 (S12)

Note that the above reformulated equation has the same form as *e^x^* = *y*/*x* whose inverse is also known as Lambert *W* function where *x* = *W*(*y*)^44^. Thus (S12) can be restated using *W*(·) as follows:

 (S13)

where the appended 0 in *W*(·) indicates the outward fluid flow. By applying square root to the both sides of (S13), *R_f_* (*t*) can be explicitly stated as:

 (S14)

Lambert *W* function was calculated using Matlab software, and the solution of (S14) was used to calculate (S10). Unlike the circular expansion scheme discussed above, the flow imbibition can also be modeled in elliptical coordinates. By letting the fluid front as *η_f_* (*t*), the flow rate in elliptical coordinate is described as^37, 45^:

$Q(t)=\frac{k}{\mu_{w}}\frac{P_{c}(\cos\psi_{1}-\cos\psi_{2})}{\frac{\eta_{f}(t)(\cos\psi_{1}-\cos\psi_{2})}{\psi_{2}-\psi_{1}}+\frac{2L_{1}}{W_{1}}}$ (S15)

where *ψ*_1_ = 0 and *ψ*_2_ = *π* for semi-elliptic flow imbibition illustrated in Supplementary Fig. S7(B). Note that plugging in *ψ*_1_ = 0 and *ψ*_2_ = *π* into (S15) yields the flow rate associated with EE model (*Q*_EE_(*t*)) below:

$Q_{\text{EE}}(t)=\frac{kHP_{c}}{\mu\left(\frac{L_{1}}{W_{1}}+\frac{\eta_{f}(t)}{\pi}\right)}$ (S16)

Calculating the fluid front *η_f_* can be done using the below relation^46^:

$\eta_{f}\sinh(2\eta_{f})-\frac{1}{2}\cosh(2\eta_{f})-\eta_{f}^{2}\cos(2\pi)+\frac{1}{2}=\frac{4kP_{c}}{a^{2}\mu_{w}}t$ (S17)

where *a* is focus of the ellipse, which is equivalent to *W*_1_/2 in semi-elliptic flow. After numerically solving (S17) using Matlab, its solution *η_f_* (*t*) was then used in (S16) for calculation.

The average flow rates were calculated over the ranges 1 ≤ *t* ≤ 15 s for CP and 1 ≤ *t* ≤ 30 s for FP, considering the pump operation times. Note that *k* can be either *k*_KB_ or *k*_CK_. While *k*_KB_ is deterministic, an appropriate *K* needs to be found for *k*_CK_. Actually, we can always tune *K* to have zero modeling error when CK permeability model is applied. However, such biased *K* was failed to yield good estimation of *Q_w_* on the other porous medium. To find an unbiased *K*, which yields fair estimates of *Q_w_* for both types of porous media, *K* was chosen to exhibit the same modeling error for each imbibition model (independent to porous media). Under the CE model, *k*_CK_ with *K* = 240 was 9.4194 × 10^-12^ m^2^ and 5.1987 × 10^-12^ m^2^ for FP and CP, respectively. Under the EE model, *k*_CK_ with *K* = 235 was 9.6199 × 10^-12^ m^2^ and 5.3093 × 10^-12^ m^2^ for FP and CP, respectively.

Overall, both CE and EE imbibition models exhibit good estimates of *Q_w_* for both KB and CK permeability models, and both FP and CP porous media as shown in Supplementary Fig. S7(C). All results are ranged within the standard deviations of the corresponding experimental values. We also compared modeling error *E_Q_* = |*Q_w_*_,Exp_ - *Q_w_*_,Mod_|/*Q_w_*_,Exp_ in Supplementary Fig. S7(D). KB model was particularly useful at estimating *Q_w_* obtained from FP; however, *E_Q_* associated with CP was higher than FP cases. As CP does not have a structured shape like FP does, this might introduce experimental variance while manually filling CP inside the pump. In the case of CK model approach, the associated *E_Q_* were the same in both FP and CP case for a given imbibition model as expected. In Fig. 2B, *Q_w_* obtained from KB model was depicted owing to its empirical-parameter-free nature and high accuracy; note that *k* given in Table 3 is equivalent to *k*_KB_.

**Note: Harkins-Brown correction factor**

Harkins-Brown (H-B) correction factor *ψ* indicates to the volume of alcohol (i.e. 3Me1Bu) droplet (*V_a_*) breakup from the nozzle to the maximum volume of alcohol that could be sustained by its surface tension (*σ_a_*), which defined as follow^48^:

$\psi=\frac{V_{a}\rho_{a}g}{\pi D_{O}\sigma_{a}}$ (S18)

where *g* is gravitational acceleration, and *ρ_a_* is density of alcohol. Since *V_a_* was unknown, the correlation between *ψ* and *ψ*/*V_a_* given in below was applied^48^:

$\psi=0.6+0.4\left[1-\frac{D_{O}}{1.46}\left(\frac{\psi}{V_{a}}\right)^{1/3}\right]^{2.3}$ (S19)

Note that utilizing (S18) and (S19) enabled us to obtain *V_a_* and *ψ*. To improve the estimation of *V_a_*, however, an empirical equation was used in the main text which referred as eq. (1)^47^.

**Note: Pumping periodicity**

To quantify the periodicity of *T*_drop_, *S*_drop_ was considered as a standard period by dividing a pump operation time with the associated *N*_drop_. The mean difference between *S*_drop_ and *T*_drop_ was denoted as *e*. For each *D_O_*, the *e*/*S*_drop_ percentage represents the irregularity of *T*_drop_ (compared to *S*_drop_) during one complete pumping cycle. We used the Weber numbers (*We* = *ρ_a_*(*U_i_*)^2^*D_O_*/2*σ_a_*) to reflect within-nozzle flow velocity (*U­_i_*) differences using the two types of porous media in Supplementary Fig. S9. The averages of all marker groups are shown as large filled markers. The extents of *T*_drop_ irregularity were similar when CP and FP were used, although *We* varied by two orders of magnitude. Given the faster flow rate associated with CP use, the CP *We* was higher than that of FP at the same *D_O_*. All averaged *e*/*S*_drop_ values were close to 7.5 %, indicating that pump operation was reasonably periodic.

**Note: Marangoni Flow Visualization**

Bromothymol blue (BTB) is a widely used pH indicator that can change its color as shown in Supplementary Fig. S10(A). Its color transition range is pH 6.0 − 7.6 in which yellow and blue color indicates acid and alkali, respectively; while green color indicates neutral pH^50^. Since very distinguishable color change is exhibited in relatively short range of pH, BTB was utilized for the visualization of Marangoni flow during the propulsion of the robot. The experimental setup is illustrated in Supplementary Fig. S10(B). Here, the experiment was conducted in a dark room where two cameras were prepared for recording in top and side views. Several lamps were installed at the bottom of the water tank to illuminate the color change of the water surface through a translucent sheet.

We adjusted the pH of the alcohol fuel as an acidic and water filled inside the water tank as alkaline according to our proposed method illustrated in Supplementary Fig. S10(C). First, a BTB solution was prepared by mixing BTB powder and ethanol (both from Daejung Chemicals) in 1:1000 weight ratio, followed by mixing the BTB solution with tap water in 1:7 weight ratio. To adjust the color as blue, sodium carbonate (Na_2_CO_3_, Daejung Chemicals) was added to set its pH value as 7.6, which was measured with a pH meter (8000 pH meter, ETI Ltd) throughout the preparation. This solution was then being filled inside the water tank up to 5 mm of height for clear visualization; increasing the water depth further degraded the clarity of color change during the recording.

To prepare an acidic alcohol fuel, 3Me1Bu, citric acid powder (Daejung Chemicals), and ethanol were mixed in 1:4:48 weight ratio. Note that ethanol was used to efficiently mix the citric acid powder with 3Me1Bu, and the pH of as-prepared acidic 3Me1Bu was 1.3. This alcohol was injected to the microchannel of the robot as propellant, and the same magnetic clamping was used to activate the embedded pump.

While the surface tension of distilled water and 3Me1Bu at room temperature is 72 mN/m and 24 mN/m, respectively, the surface tension difference between the alkaline water and the acidic 3Me1Bu is expected to be reduced because of the inclusion of other chemicals. To verify this assumption, their surface tensions were measured by pendant drop method using contact angle analyzer (Phoenix 300, SEO). After five independent measurements for each liquid, the surface tension of the alkaline water and acidic 3Me1Bu was 57.02±1.31 mN/m and 29.88±1.71 mN/m, respectively. As a result, the locomotion of the robot during the flow visualization was attenuated compared to the pristine case.

**Supplementary Figures**


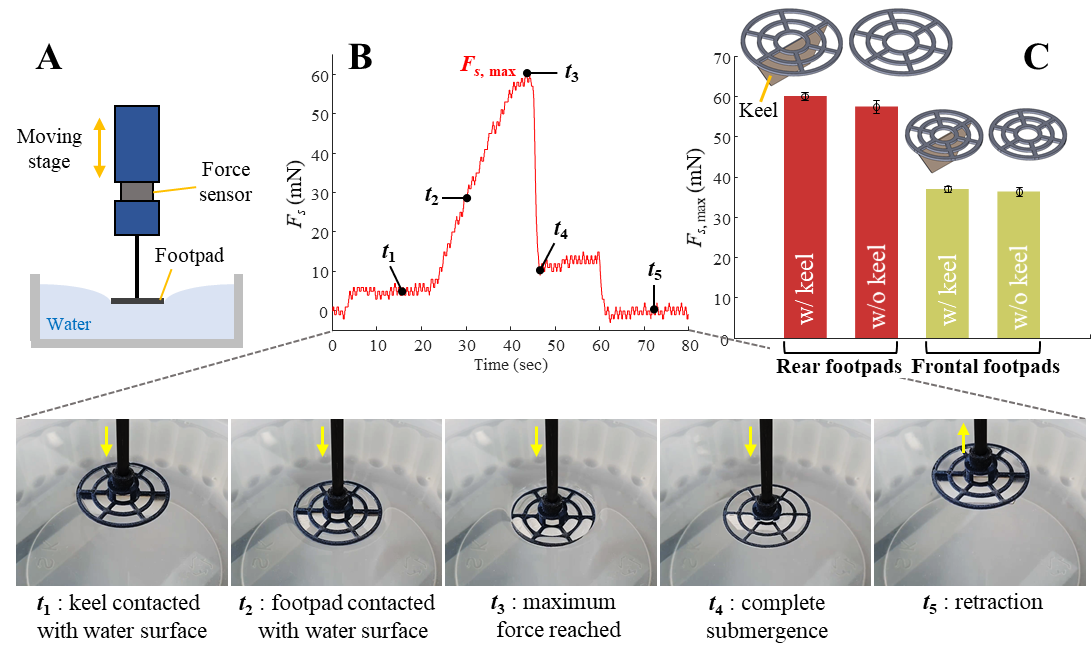


**Figure S1.** The measurement of maximal support force (*F_s_*_, max_) of the footpads. **(A)** The experimental setup for *F_s_*_,max_ measurement. **(B)** Typical support force measurement result while a footpad was slowly being contacted with water surface followed by full retraction. Note that the yellow arrows indicate the moving direction of the stage. **(C)** *F_s_*_,max_ measurement results of frontal and rear footpads with or without the extruded keel.


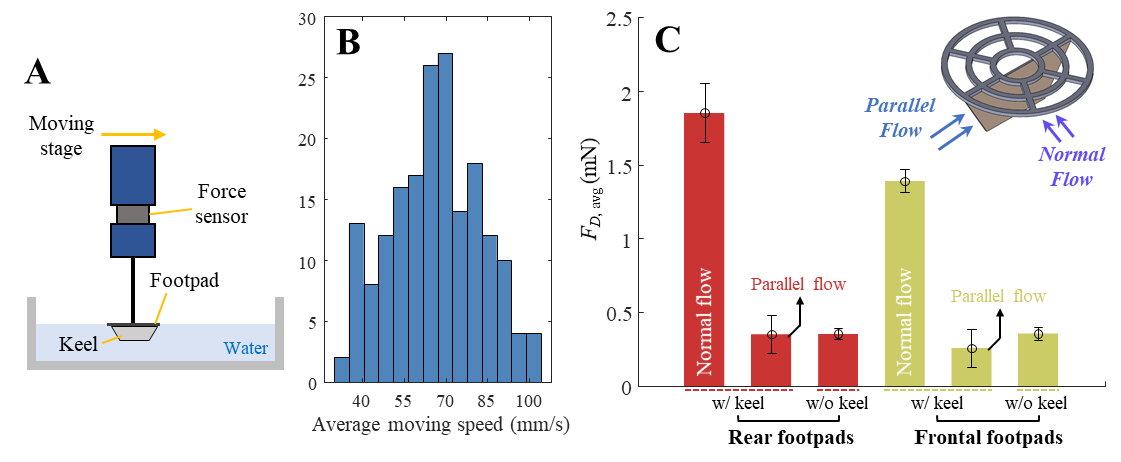


**Figure S2.** Drag force asymmetry of the proposed keel-extruded footpad. **(A)** The experimental setup for drag force measurement. **(B)** Average moving speed of the robot obtained from whole experiments to select the measurement speed. **(C)** Time averaged drag force (*F_D_*_,avg_) in parallel and normal flow directions. Note that the flow direction was not considered for the pristine (i.e. w/o keel) footpads.


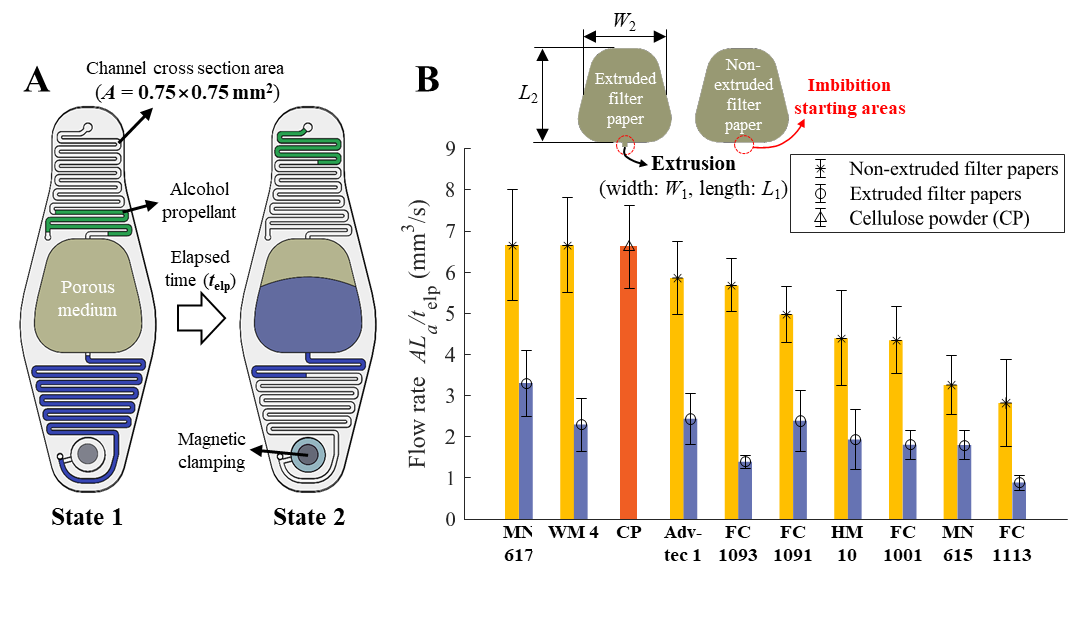


**Figure S3.** The volumetric flow rate of the propellant during the pump operation without generating any droplet. **(A)** The flow rate was measured by dividing the traveled distance of the propellant from state 1 to state 2 (*L_a_*) with elapsed time *t*_elp_ (until the frontal line of the propellant reached to the outlet) followed by the multiplication with *A*. **(B)** The comparison of flow rates; at least 7 independent measurements were performed for each porous medium.


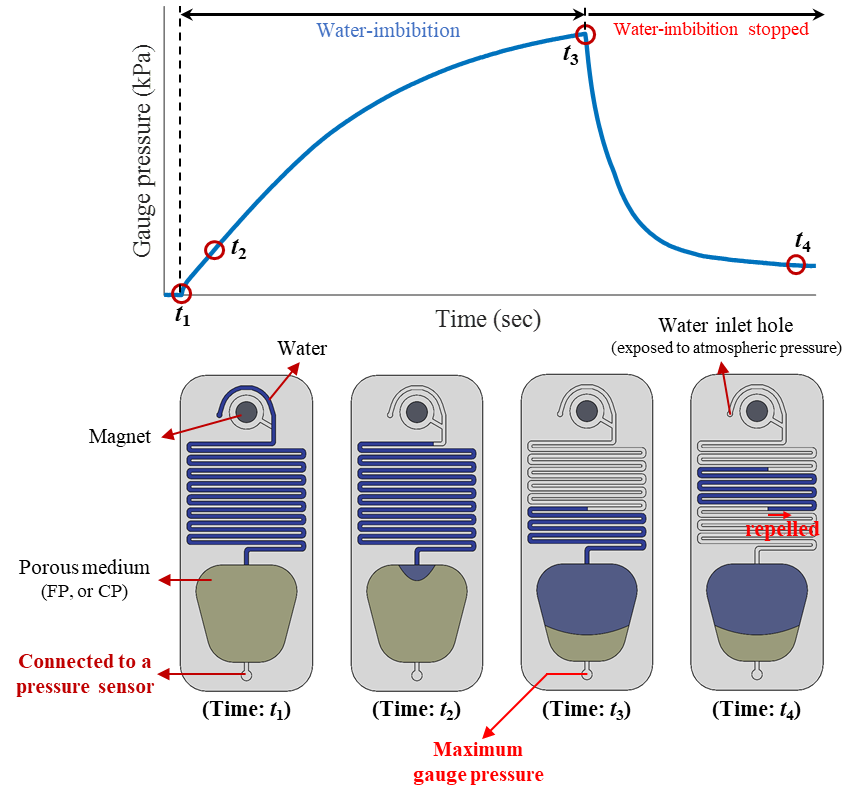


**Figure S4.** Gauge pressure measurement during water imbibition into porous media. To experimentally find capillary pressure *P_c_* that drives the flow-imbibition, the maximum gauge pressure at the terminal of a porous medium was measured (*t*_1_: after magnetic clamping, *t*_2_: earlier stage of the water imbibition, *t*_3_: the maximum gauge pressure has been reached, *t*_4_: unabsorbed water is repelled).


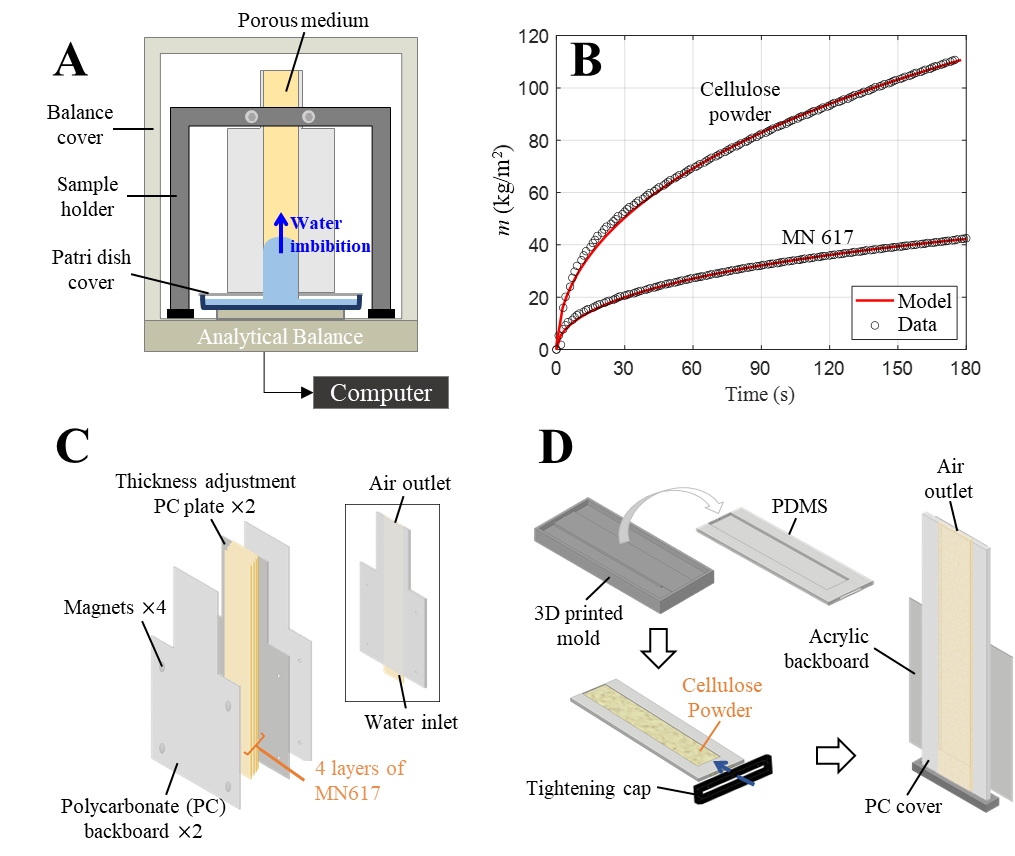


**Figure S5.** Measurement of contact angle and mean pore radius using a balance. **(A)** The schematic of a measuring apparatus. A porous medium was standing upright owing to the holder during the measurement. The weight of water contained in a petri dish started being decreased during the water-imbibition into the porous medium. **(B)** Typical experiment result of water uptake per unit area m versus time *t*, and the comparison with the solution obtained from (S4)-(S6). **(C)** The structure of MN 617 sample. Four layers of MN 617 were sandwiched between polycarbonate plates, which were then compressed with four magnets at the corners. **(D)** The structure and fabrication process of CP sample. CP was filled in a molded PDMS (Polydimethylsiloxane), and its inlet was reinforced with a tightening cap not to spill out the CP contained inside.


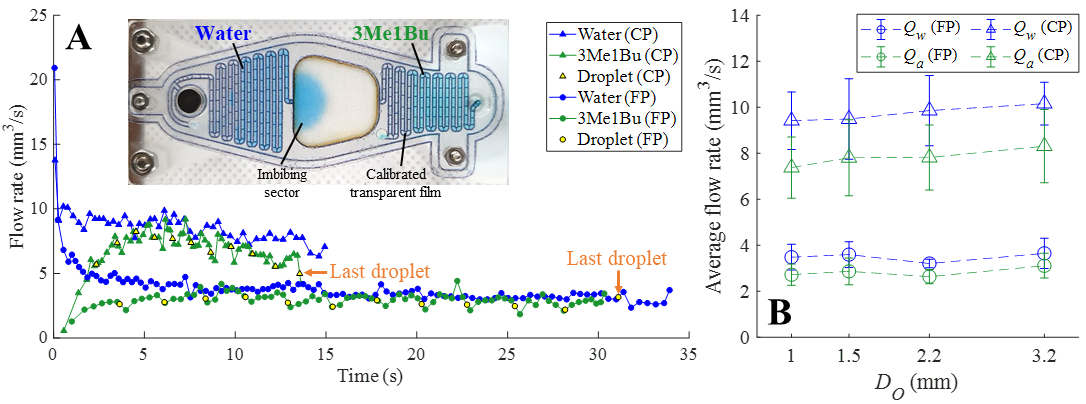


**Figure S6.** Flow rates of the pump. **(A)** Flow rates of water and alcohol (i.e. 3Me1Bu) over time during pump operation when *D_O_* = 1.0 mm. Note that the moments of alcohol droplet breakup are depicted with yellow markers. **(B)** Average flow rate of water (*Q_w_*) and alcohol (*Q_a_*).


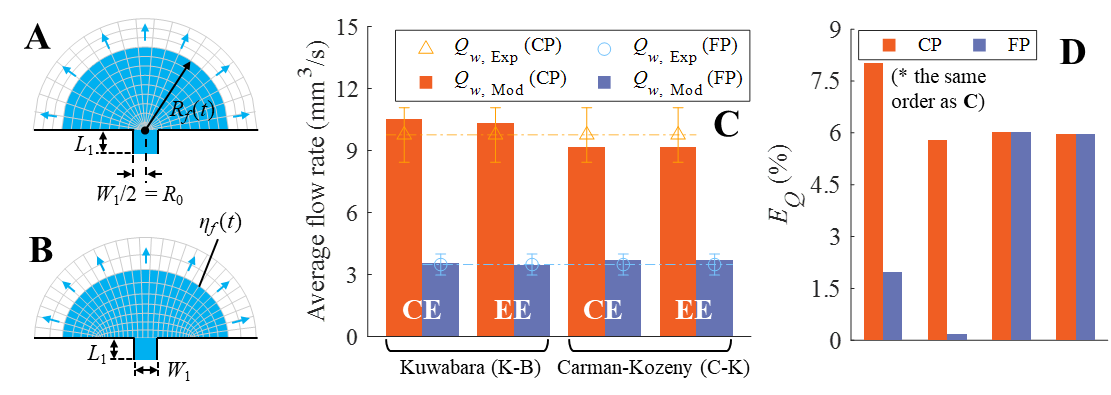


**Figure S7.** Water imbibition models of the pump. **(A)** Circular expansion (CE) model. **(B)** Elliptical expansion (EE) model. **(C)** Calculated *Q_w_* using different permeability models (K-B: Kuwabara, C-K: Carman-Kozeny) where the subscript Exp and Mod specifies the acquirement from experiment and model, respectively. **(D)** Modeling error *E_Q_* = |*Q_w_*_,Exp_ − *Q_w_*_,Mod_|/*Q_w_*_,Exp_.


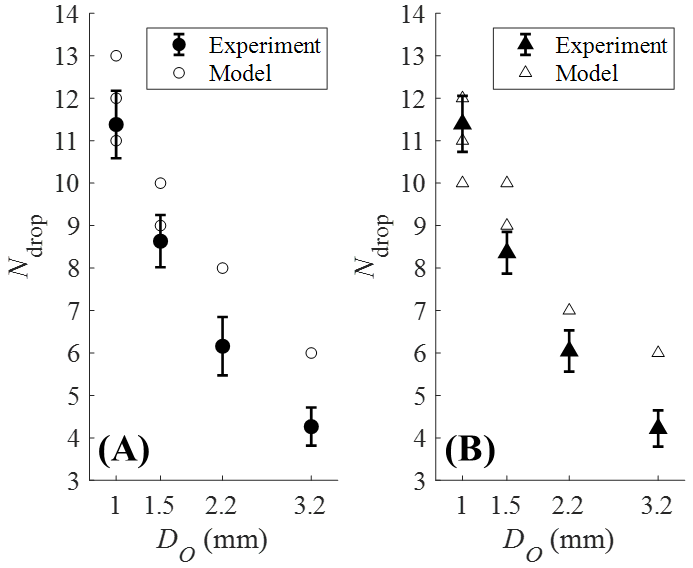


**Figure S8.** The comparison of theoretical *N*_drop_ and experimental *N*_drop_. **(A)** When MN 617 filter paper was used. **(B)** When cellulose was used. Please refer Fig. 2A and the associated main text for the definition of *N*_drop_.


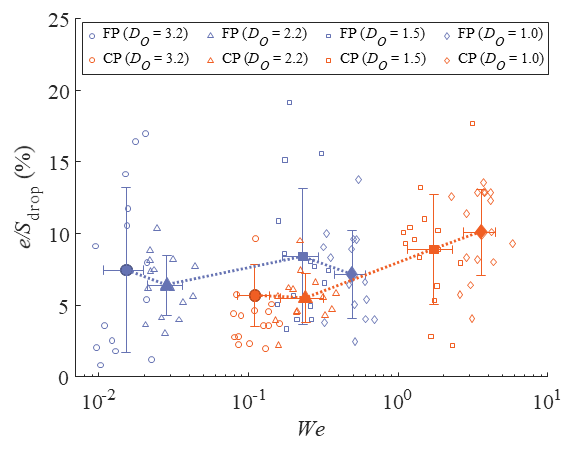


**Figure S9.** Irregularity of *T*_drop_ versus Weber number (*We*). To evaluate the periodicity of alcohol droplets generation, *e*/*S*_drop_ was considered. Please refer supplementary Note (Pumping periodicity) for more details.


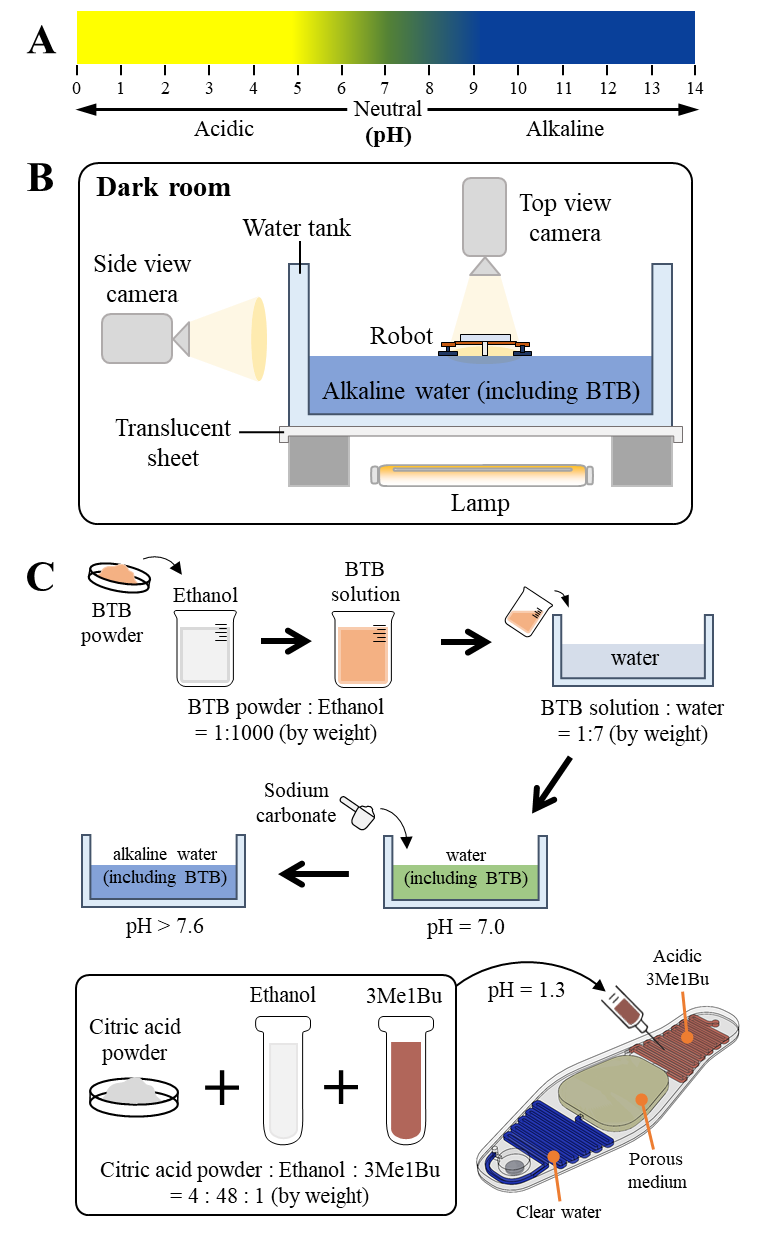


**Figure S10.** The experimental setup and method of Marangoni flow visualization. **(A)** The color change of bromothymol blue (BTB) upon pH. **(B)** The schematic of experiment environment for Marangoni flow visualization during the propulsion of the robot. **(C)** The procedures of making BTB-included alkaline water and acidic 3Me1Bu.

**
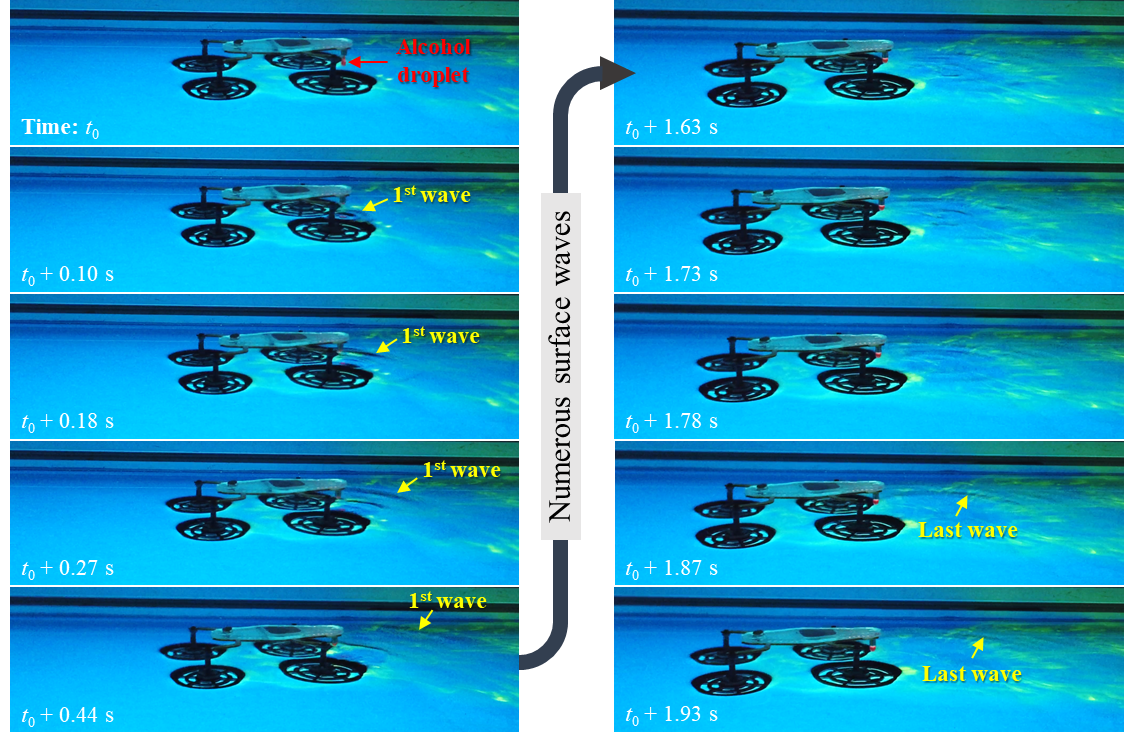
**

**Figure S11.** Marangoni flow visualization and propagation of surface waves. After one alcohol droplet was coalesced to water surface, numerous surface waves were radially propagated while propelling the robot where *D_O_* = 3.2 mm, porous medium: CP, *t*_0_: reference time.

**
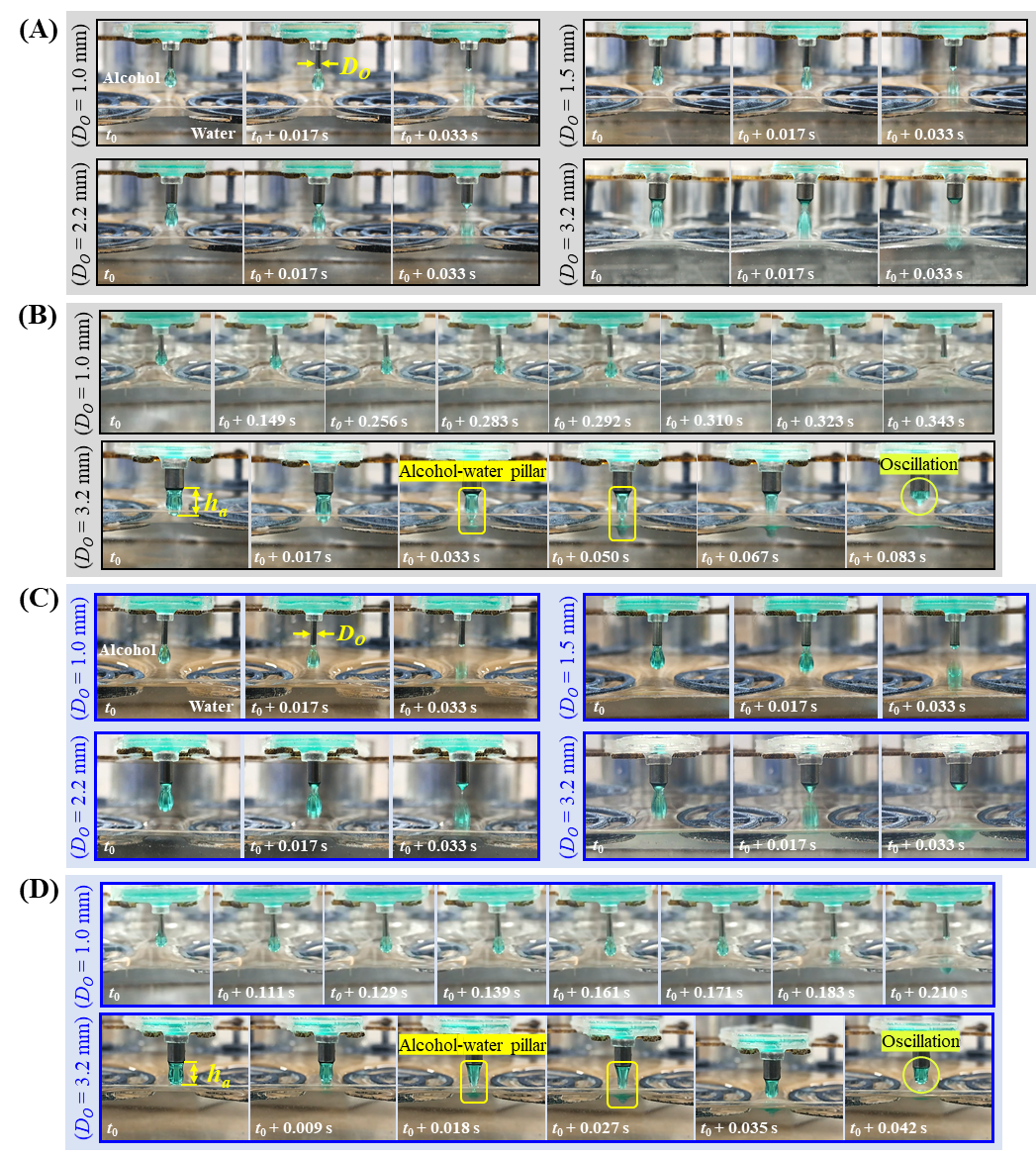
**

**Figure S12.** The sequence of alcohol droplets breakup. **(A, C)** Complete droplet breakup when *h_f_* = 9.5 mm where *t*_0_ is reference time (porous medium selection; **A**: FP, **C**: CP). **(B, D)** Incomplete droplet breakup when *h_f_* = 6.5 mm. Alcohol-water pillar was momentarily formed except *D_O_* = 1.0 mm, and the case when *D_O_* = 3.2 mm is shown as a representative (porous medium selection; **B**: FP, **D**: CP).


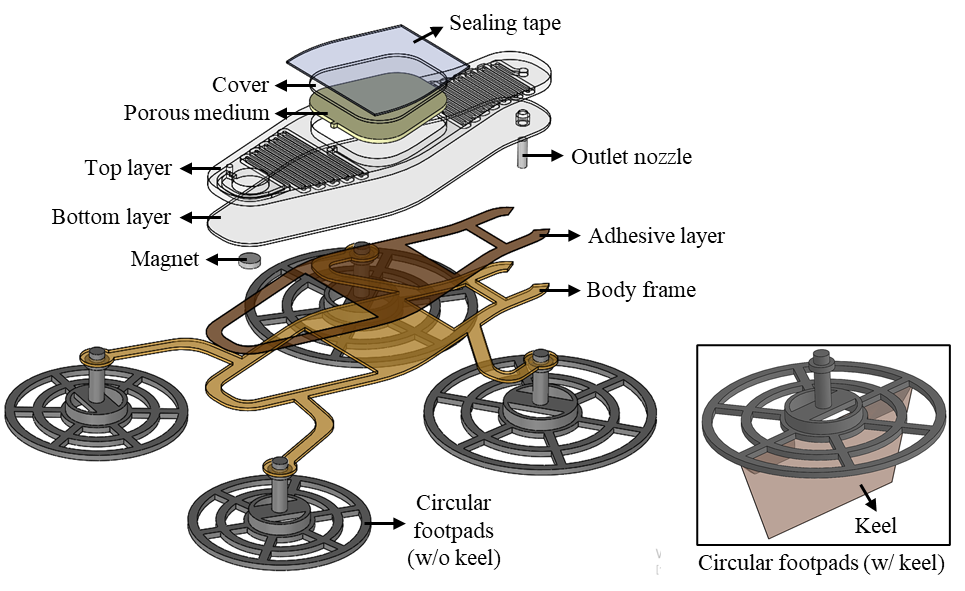


**Figure S13.** Exploded view of the proposed robot and its components. The components are made of following materials. (cover, body frame): laser-cut polycarbonate plate, (top and bottom pump layers): polydimethylsiloxane, (outlet nozzle): stainless steel tube, (magnet): neodymium, (circular footpads): 3D printed acrylonitrile butadiene styrene (ABS) followed by spray coating for hydrophobicity. Please refer Materials and Methods section for more details.

| **Filter**  **Paper** | **The number of layers used**  **In Fig. S4** | **Total**  **Thickness (mm)** | **Particle**  **Retention (μm)** | **Basis weight (g/m^2^)** | **Manufacturer** |
| --- | --- | --- | --- | --- | --- |
| MN 617 | 4 | 0.79 | 7 – 12 | 85 | Macherey- Nagel |
| MN 615 | 5 | 0.68 | 4 – 12 | 70 |  |
| WM 4 | 4 | 0.80 | 20 – 24 | 96 | Whatman |
| Advtec 1 | 4 | 0.77 | 6 | 90 | Advantec MFS |
| HM 10 | 5 | 0.67 | 6 – 10 | 70 | Hyundai Micro |
| FC 1093 | 5 | 0.77 | 35 – 40 | 80 | Chmlab |
| FC 1091 | 4 | 0.80 – 0.86 (corrugated) | 34 – 42 | 64 |  |
| FC 1001 | 5 | 0.73 | 10 – 13 | 85 |  |
| FC 1113 | 2 | 0.92 – 1.04 (corrugated) | 60 – 65 | 160 |  |

**Table S1.** The specification of commercial filter papers considered in Supplementary Figure S3. The number of layers (2nd column) refer the number of vertically stacked (laser-cut) filter papers used in the experiment to measure the volumetric flow rate in Supplementary Figure S3. The total thickness was measured by stacking the corresponding filter papers according to the number of layers in used. Note that FC 1091 and FC 1113 were corrugated, and their thickness measurement ranges were indicated. The particle retention and basis weight were obtained from the manufacturers’ datasheet. Also, the main material of the filter papers was cellulose.

| *D_O_* (mm) | The volume of  injected water (mL) | The volume of injected alcohol (mL) |
| --- | --- | --- |
| 1.0 | 0.13 ± 0.004 | 0.10 ± 0.012 |
| 1.5 |  | 0.11 ± 0.008 |
| 2.2 |  | 0.12 ± 0.005 |
| 3.2 |  | 0.14 ± 0.007 |

**Table S2.** The volume of injected water and alcohol in the pump. The volume of injected water was the same for all the sizes of outlet diameters (*D_O_*). Whereas the increased internal volume of the SUS tube at large *D_O_* connected to the outlet allowed more alcohol to be injected. Note that the height of all the SUS tubes were the same as 5 mm.

| *w_f_*  (mm) | *V*_max_ (mm/s) | *m*_robot_ (g) | m_fuel_ (g) | *ε_K_*_,max_ (μJ/g) | *We*_max_ | *Re*_max_ | Refs |
| --- | --- | --- | --- | --- | --- | --- | --- |
| 9 | 139.83 | 6.91 (w/ keel) | 0.0810  (*D_O_* = 1.0 mm) | 834 | 2.4367 | 1253 | This  work |
|  | 131.65 |  | 0.0867 (*D_O_* = 1.5 mm) | 691 | 2.1600 | 1180 |  |
|  | 146.02 |  | 0.0923 (*D_O_* = 2.2 mm) | 798 | 2.6572 | 1309 |  |
|  | 133.27 |  | 0.1094 (*D_O_* = 3.2 mm) | 561 | 2.2135 | 1195 |  |
|  | 189.84 | 6.15 (w/o keel) | 0.0810 (*D_O_* = 1.0 mm) | 1368 | 4.4914 | 1702 |  |
|  | 162.20 |  | 0.0867 (*D_O_* = 1.5 mm) | 933 | 3.2787 | 1454 |  |
|  | 160.57 |  | 0.0923 (*D_O_* = 2.2 mm) | 859 | 3.2132 | 1439 |  |
|  | 166.02 |  | 0.1094 (*D_O_* = 3.2 mm) | 775 | 3.4350 | 1488 |  |
| 10 | 148 | *ε_K_*_, max_ is adapted from (12) | | 55.1 | 3.0331 | 1474 | (12) |
| 5 | 157 |  |  | 61.6 | 1.7066 | 782 |  |
| 3 | 216 |  |  | 117 | 1.9382 | 645 |  |
| 1 | 250 |  |  | 157 | 0.8654 | 249 |  |
| 0.5 | 341 |  |  | 290 | 0.8051 | 170 |  |
| 0.3 | 385 |  |  | 371 | 0.6158 | 115 |  |
| 0.1 | 408 |  |  | 417 | 0.2305 | 41 |  |
| 30 | 79 | 0.0540 | 0.0222 | 7.5904 | 2.5926 | 2361 | (11) |
| 3.7 | 310 | *ε_K_*_, max_ is adapted from (12) | | 125 | 4.9237 | 1142 | (7) |
| 1 | 2.25 |  |  | 0.00005 | 0.00007 | 2.24 | (8) |
| 4.3 | 330 | 0.0195 | 0.001053 | 1008 | 6.4842 | 1413 | (20) |
| 10 | 105 | 0.01084 | 0.005 | 11.9511 | 1.5267 | 1046 | (9) |
| 105 | 4.5 | 39.4 | 1.578 | 0.2528 | 0.02944 | 471 | (15) |
| 50 | 27 | 0.62 | 0.01973 | 11.4541 | 0.5047 | 1345 | (24) |
| 6 | 50 | 0.02 | 0.002 | 12.5 | 0.2077 | 299 | (25) |
| 18 | 42 | 20.876 | 2.376 | 7.7494 | 0.4397 | 753 | (16) |
| 9 | 90 | 16.987 | 2.187 | 31.4574 | 1.0095 | 807 | (17) |
| 9 | 100 | 13.025 | 2.025 | 32.1605 | 1.2463 | 896 | (18) |
| 0.1 | 600 | N/A (water strider) | | | 0.4985 | 60 | (2, 53, 54) |
| 1.5 | 350 | N/A (Fisher spider) | | | 2.5444 | 523 |  |
| 0.05 | 100 | N/A (Mesovelia) | | | 0.006924 | 5 |  |
| 0.09 | 50 | N/A (Hydrometra) | | | 0.003116 | 4.48 |  |
| 0.04 | 10 | N/A (Anurida) | | | 0.000055 | 0.40 |  |
| 0.2 | 200 | N/A (Ant) | | | 0.1108 | 40 |  |
| 0.03 | 150 | N/A (Microvelia) | | | 0.009347 | 4.48 |  |
| 0.05 | 750 | N/A (Rove beetle) | | | 0.3895 | 37.35 | (56, 57) |
| 0.367 | 800 | N/A (Water beetle) | | | 3.2524 | 292.43 | (55) |

**Table S3.** The specific parameters used to evaluate *ε_K_*_,max_, *Re*_max_, and *We*_max_ in Fig. 7. The physical properties of water at room temperature (*ν_w_* = 1.004 × 10^-6^ m^2^/s, *σ_w_* = 72 × 10^-3^ N/m, *ρ_w_* = 997 kg/m^3^) were used to calculate *Re*_max_ and *We*_max_. Other parameters were directly obtained from the associated literatures. If a self-propulsion system does not have a foot, then its body width was considered as *w_f_* instead. As the explicit *ε_K_*_,max_ values of Refs. (7, 8) were available from Ref. (12) (rather than from the original references), they were employed in this table.

Movie S1 (separate file). Alcohol droplets generation when *D_O_* = 1.0 mm.

Movie S2 (separate file). Alcohol droplets generation when *D_O_* = 1.5 mm.

Movie S3 (separate file). Alcohol droplets generation when *D_O_* = 2.2 mm.

Movie S4 (separate file). Alcohol droplets generation when *D_O_* = 3.2 mm.

Movie S5 (separate file). Marangoni flow visualization during the locomotion.

Movie S6 (separate file). Formation of alcohol-water pilar.

Movie S7 (separate file). Complete and incomplete droplet break-off.

Movie S8 (separate file). Locomotion of the robot on water surface.
